# Supplementary material for: In-utero exposure to PM2.5 and adverse birth outcomes in India: Geostatistical modelling using remote sensing and demographic health survey data 2019–21
Source: PLOS Glob Public Health. 2025 Jul 2;5(7):e0003798. doi: 10.1371/journal.pgph.0003798 (PMC12220995; doi:10.1371/journal.pgph.0003798)
Supplement: S1 Table — (DOCX) [file pgph.0003798.s001.docx]

S1 Table: The weighted prevalence of low birth weight and preterm birth across Indian states and union territories, 2019-21.

| State | PTB (%) | LBW (%) |
| --- | --- | --- |
| Jammu & Kashmir | 6.08 | 10.69 |
| Himachal Pradesh | 38.66 | 15.75 |
| Punjab | 14.08 | 22.42 |
| Chandigarh | 8.78 | 16.74 |
| Uttarakhand | 26.84 | 17.90 |
| Haryana | 13.22 | 20.54 |
| Delhi | 17.43 | 21.58 |
| Rajasthan | 18.29 | 17.66 |
| Uttar Pradesh | 14.47 | 20.17 |
| Bihar | 15.99 | 16.76 |
| Sikkim | 6.41 | 9.84 |
| Arunachal Pradesh | 16.13 | 10.71 |
| Nagaland | 17.79 | 4.74 |
| Manipur | 2.70 | 7.23 |
| Mizoram | 2.00 | 4.02 |
| Tripura | 4.04 | 19.88 |
| Meghalaya | 6.57 | 11.73 |
| Assam | 12.64 | 16.20 |
| West Bengal | 12.43 | 18.95 |
| Jharkhand | 8.52 | 15.58 |
| Odisha | 4.89 | 19.13 |
| Chhattisgarh | 10.21 | 15.83 |
| Madhya Pradesh | 14.84 | 20.59 |
| Gujarat | 10.82 | 18.51 |
| Dadra & Nagar Haveli | 14.53 | 20.84 |
| Maharashtra | 8.72 | 20.06 |
| Andhra Pradesh | 6.30 | 16.22 |
| Karnataka | 15.22 | 15.87 |
| Goa | 9.34 | 14.03 |
| Lakshadweep | 3.65 | 9.73 |
| Kerala | 8.63 | 16.32 |
| Tamil Nadu | 6.25 | 16.98 |
| Puducherry | 6.55 | 13.72 |
| Andaman & Nicobar Island | 18.42 | 17.41 |
| Telangana | 14.43 | 13.92 |
| Ladakh | 8.63 | 11.61 |
| Total | **12.88** | **18.23** |
