# Supplementary material for: In-utero exposure to PM2.5 and adverse birth outcomes in India: Geostatistical modelling using remote sensing and demographic health survey data 2019–21
Source: PLOS Glob Public Health. 2025 Jul 2;5(7):e0003798. doi: 10.1371/journal.pgph.0003798 (PMC12220995; doi:10.1371/journal.pgph.0003798)
Supplement: S2 Table — (DOCX) [file pgph.0003798.s002.docx]

S2 Table: Weighted percentage of low birth weight and preterm birth by background characteristics

| Determinants | **Low birth weight** | | |  | **Preterm birth** | | |
| --- | --- | --- | --- | --- | --- | --- | --- |
|  | n | Percentage | N |  | n | Percentage | N |
| **Sex of the child** |  |  |  |  |  |  |  |
| Male | 18,397 | 17.0 | 1,07,933 |  | 15,668 | 13.1 | 1,19,809 |
| Female | 19,361 | 19.5 | 99,222 |  | 14,035 | 12.7 | 1,10,781 |
| **Birth order** |  |  |  |  |  |  |  |
| 1 | 15,939 | 18.9 | 84,222 |  | 11,654 | 12.9 | 90,246 |
| 2 | 17,808 | 17.6 | 1,01,426 |  | 14,377 | 12.7 | 1,12,779 |
| 3+ | 4,011 | 18.6 | 21,507 |  | 3,672 | 13.3 | 27,564 |
| **Mother's age at delivery** |  |  |  |  |  |  |  |
| Below 20 | 5,367 | 20.5 | 26,132 |  | 3,984 | 13.7 | 29,092 |
| 20-24 | 17,172 | 18.7 | 91,918 |  | 13,280 | 13.1 | 1,01,686 |
| 25-29 | 10,231 | 16.9 | 60,406 |  | 8,269 | 12.3 | 67,059 |
| 30&above | 4,988 | 17.4 | 28,700 |  | 4,169 | 12.7 | 32,753 |
| **Place of delivery** |  |  |  |  |  |  |  |
| Home | 2,627 | 21.5 | 12,240 |  | 3,457 | 13.4 | 25,854 |
| Institutional | 35,132 | 18.0 | 1,94,915 |  | 26,246 | 12.8 | 2,04,736 |
| **Place of residence** |  |  |  |  |  |  |  |
| Rural | 27,906 | 18.6 | 1,50,143 |  | 22,369 | 13.2 | 1,69,460 |
| Urban | 9,853 | 17.3 | 57,012 |  | 7,334 | 12.0 | 61,130 |
| **Mother's body mass index** |  |  |  |  |  |  |  |
| Underweight | 8,486 | 22.0 | 38,578 |  | 5,974 | 13.6 | 43,805 |
| Normal | 21,891 | 17.7 | 1,23,336 |  | 17,824 | 12.9 | 1,38,093 |
| Overweight/obese | 6,415 | 16.0 | 40,040 |  | 5,175 | 12.1 | 42,926 |
| **Mother's education** |  |  |  |  |  |  |  |
| Illiterate/primary | 12,820 | 20.3 | 63,286 |  | 10,608 | 13.6 | 77,809 |
| Secondary | 19,900 | 18.2 | 1,09,087 |  | 14,571 | 12.5 | 1,16,801 |
| Higher | 5,039 | 14.5 | 34,782 |  | 4,523 | 12.6 | 35,980 |
| **Wealth status** |  |  |  |  |  |  |  |
| Poor | 18,303 | 20.2 | 90,418 |  | 14,195 | 13.3 | 1,06,957 |
| Middle | 7,298 | 17.4 | 41,855 |  | 5,618 | 12.5 | 45,091 |
| Rich | 12,157 | 16.2 | 74,882 |  | 9,890 | 12.6 | 78,542 |
| **Religion** |  |  |  |  |  |  |  |
| Hindu | 30,685 | 18.5 | 1,65,473 |  | 23,624 | 12.9 | 1,83,199 |
| Muslim | 5,441 | 16.8 | 32,451 |  | 4,983 | 13.3 | 37,334 |
| Others | 1,633 | 17.7 | 9,231 |  | 1,096 | 10.9 | 10,057 |
| **Cooking fuel** |  |  |  |  |  |  |  |
| Clean fuel | 17,211 | 17.0 | 1,01,329 |  | 13,675 | 12.6 | 1,08,124 |
| Solid fuel | 20,548 | 19.4 | 1,05,826 |  | 16,028 | 13.1 | 1,22,467 |
