# Supplementary material for: In-utero exposure to PM2.5 and adverse birth outcomes in India: Geostatistical modelling using remote sensing and demographic health survey data 2019–21
Source: PLOS Glob Public Health. 2025 Jul 2;5(7):e0003798. doi: 10.1371/journal.pgph.0003798 (PMC12220995; doi:10.1371/journal.pgph.0003798)
Supplement: Attachment — Submitted filename: PGPH-D-24-02116_reviewed.pdf [file pgph.0003798.s003.pdf]

PLOS Global Public Health

In-utero exposure to PM2.5 and adverse birth outcomes in India: Geostatistical modelling using remote sensing and demographic health survey data 2019-21

--Manuscript Draft--

**Manuscript Number:**	PGPH-D-24-02116
**Article Type:**	Research Article
**Full Title:**	In-utero exposure to PM2.5 and adverse birth outcomes in India: Geostatistical modelling using remote sensing and demographic health survey data 2019-21
**Short Title:**	Exposure to PM2.5 and adverse birth outcomes in India
**Corresponding Author:**	Mary Abed Al Ahad, PhD<br>University of St Andrews<br>St Andrews, UNITED KINGDOM OF GREAT BRITAIN AND NORTHERN IRELAND
**Order of Authors:**	Arup Jana
	Malay Pramanik
	Arabinda Maiti
	Aparajita Chattopadhyay
	Mary Abed Al Ahad, PhD
**Keywords:**	Air pollution; low birth weight; NFHS data; PM2.5 risk; preterm birth, spatial modeling
**Abstract:**	Rapid urbanization in India is contributing to heightened poor air quality. Yet research on the impact of poor air on adverse birth outcomes (ABOs) especially in the public health aspect is less in India. This study investigates the influence of air quality on birth weight (LBW) and preterm birth (PTB). Utilizing data from the National Family Health Survey and satellite images, the study employs various statistical analyses and spatial models to elucidate the connection between in-utero exposure to air pollution and birth outcomes, both at the individual and district levels. It was observed that approximately 13% of children were born prematurely, and 17% were born with low birth weight. Increased ambient PM2.5 concentrations during pregnancy were associated with higher odds of LBW (AOR: 1.4; 95% CI: 1.29–1.45). Mothers exposed to PM2.5 during pregnancy had a heightened likelihood of delivering prematurely (AOR: 1.7; 95% CI: 1.57–1.77) in comparison to unexposed mothers. Climatic factors such as rainfall and temperature had a greater association with ABOs. Children residing in the Northern districts of India appeared to be more susceptible to the adverse effects of ambient air pollution. Furthermore, indoor air pollution was found to be associated with LBW. Employing a distributed spline approach, the study identified a discernible upward trend in the risk of adverse birth outcomes as the level of exposure increased, particularly following an exposure level of 40 PM2.5 ug/m3. Among the spatial models employed, the MGWR spatial model exhibited the highest level of goodness of fit. In addition to addressing immediate determinants such as nutrition and maternal healthcare, it is imperative to collaboratively address distal factors encompassing both indoor and outdoor pollution to attain lasting enhancements in child health.
**Additional Information:**	
**Question**	**Response**
**Financial Disclosure**<br><br>Enter a financial disclosure statement that describes the sources of funding for the work included in this submission and the role the funder(s) played. This includes grants and any commercial funding of the	No financial assistance has been granted from any survey organization, institutional open access or any other alternative sources to carry out the research.

Powered by Editorial Manager® and ProduXion Manager® from Aries Systems Corporation

work or authors.<br><br>This statement will be typeset if the manuscript is accepted for publication.<br><br>*Please review the submission guidelines and the instructions link below for detailed requirements and guidance.*	
**Competing Interests**<br><br>On behalf of all authors, disclose any competing interests that could be perceived to bias this work.<br><br>This statement will be typeset if the manuscript is accepted for publication.<br><br>*Please review the instructions link below and PLOS Global Public Health's competing interests policy to determine what information must be disclosed at submission.*	The authors declare that they have no conflict of interest.
**Data Availability**<br><br>Before publication, Authors are required to make fully available and without restriction all data underlying their findings. Please see our PLOS Data Policy page for detailed information on this policy.<br><br>A **Data Availability Statement**, detailing where the data can be accessed, is required at first submission. Insert your Data Availability Statement in the box below.<br><br>Please see the data reporting section of our submission guidelines for instructions on what you need to include in your Data Availability Statement.	The study uses secondary data that are available on reasonable request through https://dhsprogram.com/data/dataset_admin/

This statement will be typeset if the manuscript is accepted for publication.<br><br>*PLOS allows rare exemptions to address legal and ethical concerns. If you have legal or ethical restrictions, please use the box below to detail these in full sentences for the Journal team to consider.*	

Manuscript Click here to access/download;Manuscript;manuscript (2).docx

In-utero exposure to $PM_{2.5}$ and adverse birth outcomes in India: Geostatistical modelling using remote sensing and demographic health survey data 2019-21

Arup Jana[1], Malay Pramanik[2], Arabinda Maiti[3] and Aparajita Chattopadhyay[4], Mary Abed Al Ahad[5]*

*Corresponding author

LIST OF AUTHORS: NAME, AFFILIATION & ADDRESS

1. Research Associate, Indian Institute of Technology Delhi, Hauz Khas, New Delhi - 110 016, India
2. Urban Innovation and Sustainability, School of Environment, Resources and Development (SERD), Asian Institute of Technology (AIT), P.O. Box 4, Klong Luang, Pathumthani 12120, Thailand
3. Postdoctoral Fellow, School of Geography, Newman Building, University College Dublin, Belfield, Dublin 4, Ireland
4.Department of population & Development, International Institute for Population Sciences, Mumbai,40088, India.
5. Associate Lecturer, School of Geography and Sustainable Development, University of St Andrews, Irvine Building, KY16 9AL, St Andrews, Scotland, United Kingdom. Email: maaa1@st-andrews.ac.uk

The authors declare that they have no conflict of interest.

Graphical abstract:

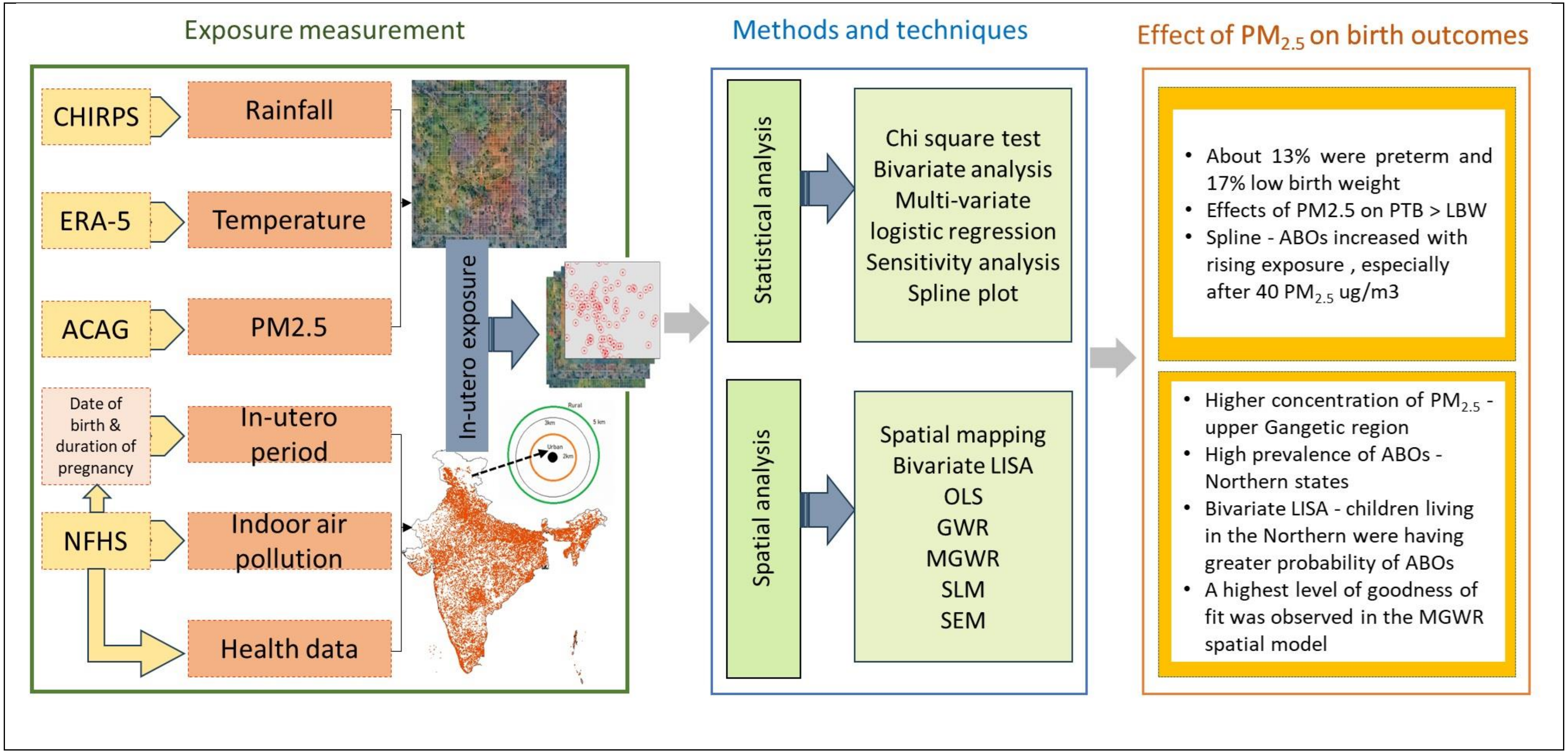

In-utero exposure to PM2.5 and adverse birth outcomes in India: Geostatistical modelling using remote sensing and demographic health survey data 2019-21

Abstract:

Rapid urbanization in India is contributing to heightened poor air quality. Yet research on the impact of poor air on adverse birth outcomes (ABOs) especially in the public health aspect is less in India. This study investigates the influence of air quality on birth weight (LBW) and preterm birth (PTB). Utilizing data from the National Family Health Survey and satellite images, the study employs various statistical analyses and spatial models to elucidate the connection between in-utero exposure to air pollution and birth outcomes, both at the individual and district levels. It was observed that approximately 13% of children were born prematurely, and 17% were born with low birth weight. Increased ambient $PM_{2.5}$ concentrations during pregnancy were associated with higher odds of LBW (AOR: 1.4; 95% CI: 1.29–1.45). Mothers exposed to $PM_{2.5}$ during pregnancy had a heightened likelihood of delivering prematurely (AOR: 1.7; 95% CI: 1.57–1.77) in comparison to unexposed mothers. Climatic factors such as rainfall and temperature had a greater association with ABOs. Children residing in the Northern districts of India appeared to be more susceptible to the adverse effects of ambient air pollution. Furthermore, indoor air pollution was found to be associated with LBW. Employing a distributed spline approach, the study identified a discernible upward trend in the risk of adverse birth outcomes as the level of exposure increased, particularly following an exposure level of 40 $PM_{2.5}$ ug/m^3. Among the spatial models employed, the MGWR spatial model exhibited the highest level of goodness of fit. In addition to addressing immediate determinants such as nutrition and maternal healthcare, it is imperative to collaboratively address distal factors encompassing both indoor and outdoor pollution to attain lasting enhancements in child health.

Keywords: Air pollution; low birth weight; NFHS data; $PM_{2.5}$ risk; preterm birth, spatial modeling.

Highlights:

- An estimated 13% of the children were preterm and 17% were low birth weight in India in 2019-21.
- High $PM_{2.5}$ concentration during pregnancy was associated with LBW and PTB.
- Children residing in the Northern districts of India were found to be more vulnerable to poor air quality.
- A rising trend in the risk of adverse birth outcomes was observed after exposure level of 40 $PM_{2.5}$ ug/m3.
- The MGWR spatial model demonstrated the highest level of goodness of fit.

1. Introduction

Ambient air pollution poses an existential global environmental threat to planetary and human health, with a disproportionate burden of its detrimental effects falling on those residing in low and middle-income countries [1]. Consequently, the United Nations Climate Change Conference has urged developed countries to provide financial support to less developed and developing countries to confront the adverse impacts of air pollution and establish appropriate mechanisms to mitigate climate change [2]. Besides being a significant driver of climate change, air pollution is the most critical risk factor for adverse health consequences [3]. Referred to as the "silent killer," ambient air pollution is among the top five risk factors for mortality in both males and females [4–6]. In 2019 alone, ambient particulate matter pollution was responsible for 118 million disability-adjusted life years (DALY) and 4.14 million deaths in 2019 [7]. Among air pollutants, ambient fine particulate matter ($PM_{2.5}$) is considered the most harmful air pollutant [4, 8–10]. These particles primarily originate from the burning of fossil fuels and biomass [11]. With a diameter of less than 2.5 microns, exposure to these particles increases the risk of respiratory diseases, lung cancer, stroke, and heart disease [9,12–16].

In the 2023 World Air Quality Report, India was ranked as the third most polluted country out of 134 nations based on its average yearly PM2.5 levels [17]. Notably, about 7 out of 10 Indians are exposed to air pollution levels that exceed the national standard of 40 µg/m3 [18]. Alarmingly, in 2019, as many as 0.98 million deaths in India were attributed to ambient particulate matter pollution [19]. The key factors of this alarming trend are primarily attributed to the rising air pollution levels in the country due to urbanization and industrialization [1,20,21]. To address this critical air pollution situation, the government of India introduced the National Clean Air Program in 2019, setting a targeted reduction in air pollution. It envisages reaching a minimum 20% reduction in particulate matter concentration by 2024, compared to 2014 levels [22,23]. However, despite these significant efforts, India still ranks as the second most polluted country in the world [22].

While air pollution has adverse impacts on the health of individuals across all age groups, infants and children are considered more vulnerable due to their developing organs and higher air intake per unit of body weight [24,25]. Ambient air pollution has been associated with a range of pediatric morbidities, including adverse birth outcomes (ABO), asthma, cancer, and an increased risk of chronic diseases in life stages [9,13,26–30]. Among the ABOs associated with ambient air pollution are preterm birth (PTB) and low birth weight (LBW) [25,27,31,32]. PTB and LBW are important predictors of under-five mortality and malnutrition [33,34]. The World Health Organization estimates that approximately 15 million babies are born preterm (<37 weeks) each year [34]. Furthermore, 15% of babies worldwide are born with LBW, defined as <2500 grams at birth [35]. While LBW is a global public health concern, its prevalence is particularly higher in low and middle-income countries [36].

A systematic review of 41 studies found that exposure to $PM_{2.5}$ is associated with PTB, LBW, and small-for-gestational-age births [37]. A prospective cohort study conducted in Tamil Nadu provided the first quantitative evidence linking rural-urban $PM_{2.5}$ exposures during pregnancy of LBW [38]. This study concluded that increased exposure to particulate matter exposure during pregnancy is associated with a decrease in birth weight among newborns [39]. However, research in developing countries, albeit limited, established that maternal exposure to ambient air pollution is associated with higher odds of LBW and PTB among children [40–43].

The majority of studies investigating the association between ambient air pollution with ABOs have primarily been conducted in high-income countries [36,40]. The evidence regarding this particular issue is somewhat less in developing countries [32]. According to National Family Health Survey-5, 18% of children born in the five years

preceding the survey had low birth weight. India has been identified as a significant contributor to global preterm births [44]. Moreover, ambient air pollution levels are expected to increase in India as the country is transitioning from a rural economy to an urban one, with the urban population projected to reach 53% by 2050 from 35.87 in 2022 [45]. Despite the alarming rise in air pollution levels in India, there has been a paucity of research exploring its impact on ABOs. Moreover, the findings of a spatial analysis on the effects of air pollution on adverse birth outcomes (ABOs) will be helpful for implementing area-specific schemes for policymakers or stakeholders, which has not yet been done at the national level in India. Therefore, the study hypothesizes a negative impact of $PM_{2.5}$ on birth outcomes in India. In order to address this research gap in the available evidence, the present study endeavors to investigate the impact of ambient air pollution on ABOs, particularly focusing on LBW and PTB at the national level. Further, the study utilized different geospatial models to highlight the vulnerable areas that need to be focused on, with robust estimated outcomes controlling for environmental, demographic, and socioeconomic confounding factors. The study utilizes nationally representative data from the latest round of the NFHS, 2019-21, thus ensuring the generalizability of results [46]. The results of this study might be imperative as LBW and PTB continue to be significant public health concerns in India. Moreover, this study will augment the existing but limited literature focused on investigating the association of ambient air pollution with LBW and PTBs, a question of paramount public health significance.

2.Data and Methods

2.1. Population and health data

Population data were extracted from the fifth NFHS, conducted across 36 states and union territories at the national level. A stratified two-stage sampling procedure was employed in the survey design. Primary Sampling Units (PSUs) were villages in rural areas and census enumeration blocks (CEBs) in urban areas. Survey questionnaires were prepared to take out detailed information on maternal and child health, as well as socioeconomic data. Additionally, the NFHS data incorporated the collection of biomarker data [46]. The survey encompassed reproductive history, including data on contraceptive use, pregnancy, birth, termination, and abortion. Children born 0 to 5 years preceding the survey were considered in the research. The protocol for the NFHS-5 survey, including the content of all the survey questionnaires, received approval from both the International Institute of Population Sciences (IIPS) institutional review board and the ICF institutional review board. Furthermore, it underwent review protocol by the U.S. Centres for Disease Control and Prevention (CDC). Further details on the sampling design can be obtained in the Indian National Report (https://dhsprogram.com/pubs/).

2.2. Air pollution data

In this study, global level $PM_{2.5}$ data was derived from the Atmospheric Composition Analysis Group (ACAG), to measure the exposure of mothers during the pregnancy period [47]. The spatial resolution of $PM_{2.5}$ was $0.01° \times 0.01°$ and Resolution-Tiered Approach (RTA) was employed to estimate the concertation of $PM_{2.5.}$ The data is prepared by the fusion of multiple sensors including Aerosol Optical Depth (AOD) measurements from NASA's Moderate–Resolution Imaging Spectro-radiometers (MODIS), Multi-Resolution Imaging Spectro-Radiometers (MISR), and Sea–viewing Wide Field–of–View Sensor (SeaWiFS) instruments with the Goddard

Earth Observing System (GEOS) and chemical transport model (Chem) data. The data was further calibrated to match global ground-based observations using a Geographically Weighted Regression (GWR) technique, and its accuracy has been validated in several previous studies [43,48–55]. Each survey participant was assigned an average in-utero air pollution exposure based on the child's date of birth and the duration of pregnancy, as recorded in the NFHS survey data. For instance, if a child was born in January 2019 with a pregnancy duration of 9 months, the in-utero exposure period was considered from May 2018 to the end of January 2019. The cluster points were randomly displaced by 2 and 5 km in urban and rural areas, respectively, to maintain the privacy of participants. Thus, a 3 km buffer was created around each cluster point for the extraction of pollutant data.

2.3 Climate data

We used the Climate Hazards Group InfraRed Precipitation with Station (CHIRPS) precipitation dataset, covering a span of 43 years from 1981 to the present [56], with a spatial resolution of 5 km (https://www.chc.ucsb.edu/data/chirps). This high-quality dataset served as the foundation for developing a comprehensive and robust climatic index, laying the groundwork for deeper insights into the long-term trends and patterns of climatic hazards in various geographical areas.

We also utilized the ERA5-Land product, distributed by the Copernicus Climate Change Service (C3S) Climate Data Store (CDS) of ECMWF, to analyze land surface temperature (LST) in our study [57]. This dataset provides monthly temperature values at a spatial resolution of 0.1 degrees (approximately 11,200 meters at the equator) on a latitude/longitude Climate Modelling Grid (CMG), covering the period from January 1981 onwards. The ERA5-Land product, along with its fine resolution and long-term availability, enables detailed assessments of LST variations and their implications for various ecosystems and human activities [56,58–60].

2.4 Variable description

2.4.1. Dependent variables

According to the World Health Organization (WHO), Preterm birth is defined as a live birth before 37 completed weeks of gestation, while low birth weight is defined as weight at birth less than 2500 grams [61]. In the present study, we followed the methodology outlined by Jana, Banerjee, and Khan (2023) [62], to measure preterm birth using the calendar method of the Demographic and Health Surveys (DHS). The NFHS collected data on birth weight using the following questions: Was (name of the child) weighed at birth? How much did (name of the child) weigh? The information was reported in two ways; first, the mother recalled about her baby's weight, and second, reported with the help of any card of their baby's weight [46]. The other outcome variable in this study was preterm birth, which is estimated based on the duration of pregnancy. Both outcome variables are dichotomous, with '0' signifying that the child did not have LBW/was not preterm, and '1' indicating that the child had LBW/was preterm.

2.4.2. Model covariates

We selected the factors associated with adverse birth outcomes based on published literature and the availability of variables within the dataset. The study included the child's characteristics, such as the sex of the child (male

and female) and birth order (1st, 2nd and 3rd & above), which were considered as confounding factors. Babies born at home was defined as non-institutional delivery and delivery taken place at any medical institution was considered as institutional delivery. Mother's general health plays a pivotal role in determining the health of newborns [63,64]. NFHS-5 collected anthropometric measurements using biomarkers. These measurements were used to calculate Body Mass Index (BMI) by dividing weight in kilograms by height in meters squared (kg/m^2). BMI was categorized into four groups; thin (BMI <18.5), normal (BMI 18.5–24.9), overweight (BMI 25–30), and obese (BMI≥30.0).

The model incorporated several maternal variables, such as mother's level of education (categorized as illiterate/primary, secondary, and higher education) and mother's age at the time of childbirth (< 20, 20 – 24, 25-29, and 30 years & above). In the survey, respondents were asked about their primary cooking fuel source. We classified wood, coal, dung cake, and crop residue as solid or unclean fuel and natural gas, liquefied petroleum gas, and electricity as clean fuels. The household’s wealth quintile was categorized into three categories; 'poor', 'middle' and 'rich'. Additionally, the household’s religion was recoded as 'Hindu', 'Muslim' and 'Others' (such as Sikh, Christian, Jain etc.).

2.5. Statistical analysis

The weighted prevalence of LBW and PTB was estimated using the exposed sample, and the Chi-square test ($\chi 2$) was performed to evaluate the association between dependent and independent variables. Geospatial analysis has been conducted using ArcGIS (version 10.8) to prepare the prevalence maps. Further, multivariate logistic regression was employed to examine the association between exposure to $PM_{2.5}$ during pregnancy and birth outcomes after controlling all the possible confounding factors. The logistic regression model is defined as,

$$logit(p) = \log\left(\frac{p}{1-p}\right) = \beta_0 + \beta_1 * x_1 \ldots\ldots. + \beta_k * x_k + \varepsilon$$

Where, β_0 is intercept and β1… βk are regression coefficients indicating the relative effect of a particular explanatory variable on the outcome, while ε is an error term. Further, bivariate Local Indicators of Spatial Association (LISA) was used in the study to explore the spatial association between exposure to $PM_{2.5}$ and adverse birth outcomes. A sensitivity analysis was employed by adjusting different determinants to explore the relation between in-utero exposure to $PM_{2.5}$ and birth outcomes such as PTB and LBW. Further, in the study, a spline function was utilized to visually depict the connection. This connection involved the likelihood of occurrences such as having children with LBW and PTB, all influenced by exposure to air pollution during pregnancy. A spline function integrates several polynomial sections linked by knots, forming a continuous curve. The quantity of knots can be modified to control the curve and smoothness, increasing or decreasing it as needed. To determine knot placement, we employed quantiles of the exposure level of $PM_{2.5}$ in the individuals.

Bivariate LISA measures the local correlation between a variable and the weighted average of another variable in the neighborhood.

$$I_i = n_i \sum_j w_{ij} z_j$$

Where, Z_i denotes standardized variable of interest and W_{ij} is weight matrix. The bivariate LISA functionality the cluster maps of the association between two variables. The cluster map is a special choropleth map showing those locations with a significant local Moran statistic classified by the type of spatial correlation: bright red for high-high associations, bright blue for low-low, light blue for low-high and light red for high-low. The high-high and low-low suggest clustering of similar values, whereas high-low and low-high locations indicate spatial outliers. The scatter plot of the dependent and independent variables of a spatial unit shows the statistical figure of the association.

Several geostatistical models were used in this study to identify the best-fitting model, and the results will be helpful for policymakers to launch area-specific schemes for vulnerable regions. . . First, Ordinary Least Squares (OLS) technique was used. This is a classic linear regression model that seeks to find the best-fitting line through the data by minimizing the sum of squared differences between observed and predicted values. OLS assumes that the relationship between the dependent and independent variables is constant across space. Second, we employed Geographically Weighted Regression (GWR) which is a spatial regression technique that allows for the exploration of spatially varying relationships between variables. It recognizes that the relationships may change across different geographic locations, providing a localized understanding of the data. Finally, Multiscale Geographically Weighted Regression (MGWR) which extends GWR by considering relationships at multiple spatial scales was applied [65]. This model accounts for varying relationships not only at the local level but also extends its analysis across different geographic scales, providing a more comprehensive analysis of spatial variability. The model employs two spatial regression models, namely the Spatial Lag Model (SLM) and the Spatial Error Model (SEM), each addressing distinct aspects of spatial dependency. The Spatial Lag Model (SLM) is a type of spatial autoregressive model that considers the spatial dependency of observations. It assumes that the values of the dependent variable are influenced by the values of neighboring observations, incorporating spatial relationships into the model [66]. It accounts for the spatial interdependence of data points. On the other hand, the Spatial Error Model (SEM) is another spatial regression model that accounts for spatial autocorrelation in the error terms of the model. It assumes the presence of spatially correlated errors that are not captured by the independent variables, allowing for a more accurate representation of the data's spatial structure.

3. Results

3.1 Characteristics of the sample

Table 1 represents the percentage distribution of the sample size used in the present study. Approximately 52% of the sample consisted of female children, and the rest of the sample was male children. Among the sample, 13% of the sample were born preterm, whereas 17% of the sample were LBW. About half of the sample belonged to 2nd birth order category. One out of 10 mothers were teenagers (under 20 years of age). About 14% of the children were born at home, and nearly 19% of the mothers were undernutrition or categorized as 'thin'. Half of the sample belonged to households classified as 'poor', and 73% of the sample belonged to the Hindu religion. Additionally, 58% of the households reported using solid fuels for cooking.

3.2 Spatial distribution of ABO and $PM_{2.5}$

The spatial distribution of ambient $PM_{2.5}$ shows a high concentration over the upper Gangetic region (**Figure 1**), covering states like Uttar Pradesh, Bihar, Delhi, Punjab and Haryana, whereas a lower concentration was

observed in the Southern and North-Eastern regions of India. The highest prevalence of preterm was observed in the Northern states, such as Himachal Pradesh (39%), Uttarakhand (27%) and Rajasthan (18%), and Delhi (17%), including North-Eastern states like Nagaland (**Table S1**). In contrast, a lower prevalence of PTB was observed in Mizoram, Manipur and Tripura. As for LBW, the highest prevalence was in Punjab (22%), followed by Delhi, Dadra and Nagar Haveli, Madhya Pradesh, Haryana and Uttar Pradesh. Conversely, fewer children with LBW were observed in the North-East region of India (**Figures 1b and 1c**).

3.3 Effects of $PM_{2.5}$ on ABO at the individual level

Figure 2 shows the weighted percentage of LBW and PTB by selected background characteristics. The observation revealed that the prevalence of LBW was higher among females (20%) compared to males (17%), whereas a significant difference was not observed for PTB (**Table S2**). In both cases, the proportion was higher among teenage mothers. However, a decreasing percentage was found with age for LBW, but an increasing level of PTB can be observed after age 30 years. The percentage of adverse birth outcomes was greater among children born at home and residing in rural areas. Underweighted mothers had more LBW (22%) and PTB (13%) children than normal mothers, 17% and 12%, respectively. Illiterate and primary educated mothers were having more LBW and PTB children. A similar result can be observed for wealth status. Poor mothers had more adverse birth outcomes. However, mothers who belonged to the Muslim religion had a lower percentage of LBW and a higher proportion of PTB. Households using solid fuels for cooking had more adverse birth outcomes.

Our unadjusted and adjusted multivariate logistic regression models consistently revealed associations between air pollution during pregnancy and birth outcomes, as shown in **Table 2**. Higher ambient PM2.5 concentrations during pregnancy were associated with higher odds of both low birth weight (LBW) (AOR: 1.37; 95% CI: 1.29–1.45) and preterm birth (PTB) (AOR: 1.67; 95% CI: 1.57–1.77). The odds ratios were higher in the unadjusted models for both LBW (OR: 1.56; 95% CI: 1.50–1.63) and PTB (OR: 1.62; 95% CI: 1.55–1.69). A slight increase in temperature was associated with higher odds of LBW (AOR: 1.03; 95% CI: 1.01–1.04), though it was not significantly associated with PTB, while higher rainfall was significantly associated with both LBW (AOR: 1.07; 95% CI: 1.03–1.12) and PTB (AOR: 1.04; 95% CI: 1.02–0.10). The use of solid fuel for cooking was associated with higher odds of LBW (AOR: 1.04; 95% CI: 1.01–1.07), but it was not significantly associated with PTB. Female children had higher odds of being born with LBW (AOR: 1.18; 95% CI: 1.15–1.20) compared to male children but had slightly lower odds of PTB (AOR: 0.97; 95% CI: 0.95–1.00). Higher birth order was associated with lower odds of LBW (AOR for second birth: 0.89; 95% CI: 0.86–0.91; AOR for third or higher birth: 0.84; 95% CI: 0.80–0.88). For PTB, only a third or higher birth order showed a significant decrease in odds (AOR: 0.94; 95% CI: 0.89–0.98). Teenage mothers (below 20 years) had higher odds of giving birth to children with LBW (AOR: 1.09; 95% CI: 1.04–1.15) and PTB (AOR: 1.08; 95% CI: 1.02–1.14). Children born at home had higher odds of LBW (AOR: 1.11; 95% CI: 1.06–1.16). Urban residence was associated with higher odds of LBW (AOR: 1.07; 95% CI: 1.04–1.11) and lower odds of PTB (AOR: 0.96; 95% CI: 0.92–0.99). Underweight mothers had higher odds of both LBW (AOR: 1.27; 95% CI: 1.24–1.31) and PTB (AOR: 1.04; 95% CI: 1.01–1.08), while overweight or obese mothers had lower odds of both LBW (AOR: 0.93; 95% CI: 0.90–0.96) and PTB (AOR: 0.95; 95% CI: 0.92–0.99). Lower maternal education levels were associated with higher odds of LBW (AOR for illiterate/primary: 1.40; 95% CI: 1.34–1.46; AOR for secondary: 1.23; 95% CI: 1.18–1.28), while higher education was associated with lower odds of PTB (AOR: 0.95; 95% CI:

0.91–0.99). Poor mothers had higher odds of LBW (AOR: 1.09; 95% CI: 1.05–1.13) and lower odds of PTB (AOR: 0.92; 95% CI: 0.88–0.95). Muslim mothers had lower odds of LBW (AOR: 0.87; 95% CI: 0.84–0.91) and similar odds of PTB compared to Hindu mothers.

There was a high likelihood of LBW in women who experienced high levels of $PM_{2.5}$ during their gestational period (**Figure 3 a and 2b**). Employing a distributed spline approach, the study identified a growing trend in the risk of delivering LBW babies as the exposure level increased, especially after the exposure level of 40 $PM_{2.5}$ ug/m^3. Concerning PTB, the odds displayed a rapid and exponential increase in relation to the $PM_{2.5}$ exposures and the increasing trend became notably rapid after reaching the exposure level of 50 $PM_{2.5}$ ug/m^3.

3.4 Sensitivity analysis

The unadjusted odds ratio demonstrates a significantly higher likelihood of LBW associated with PM2.5 exposures during pregnancy (OR: 1.56; 95% CI: 1.50–1.63). However, the strength of this association decreased after adjusting for environmental, socioeconomic, maternal, and child characteristics (**Table 3**). In contrast, for PTB, the odds ratio shifted from 1.62 in the unadjusted model to 1.67 after accounting for these determinants. This discrepancy underscores the influence of the various determinants considered in the study on the odds ratio values, highlighting their importance in the analysis.

3.5 Spatial association between $PM_{2.5}$ and ABO

The findings of the bivariate LISA map showed that children living in the Northern districts of India faced a higher vulnerability to ambient air pollution as the high-high clusters of spatial association were found in Punjab, Delhi, Madhya Pradesh, Rajasthan and some parts of Uttar Pradesh (**Figure 4**). A total of 109 districts had a significant association between in-utero exposure to $PM_{2.5}$ and LBW. On the other hand, 40 districts had high-high clusters of autocorrelations between $PM_{2.5}$ and PTB. Notably, it was observed that most of the districts of Uttar Pradesh were found to be more vulnerable to $PM_{2.5}$ in the context of preterm birth.

Additionally, the study employed OLS, GWR, MGWR, SLM, and SEM, to investigate the spatial relationship between $PM_{2.5}$ exposures during pregnancy and the occurrences of LBW and PTB, while considering potential confounding factors. In line with our hypothesis, the analysis revealed a robust spatial association between $PM_{2.5}$ exposures and LBW, although the strength of this association varied across the different models. Notably, the GWR model showed the highest impact of $PM_{2.5}$ exposures (β=0.122, SE=0.009), while MGWR demonstrated the highest goodness-of-fit (R^2 = 68%) (**Table 4**). Regarding PTB, the MGWR model provided the best explanatory power, accounting for 54% of the variance, surpassing other models. In the GWR model, a noteworthy finding was that a one-unit increase in $PM_{2.5}$ exposure corresponded to a 5% increase in the prevalence of PTB (**Table 5**) and a 12% increase in LBW. Overall, a reduction in the prevalence of LBW and PTB was observed across all models, with MGWR and GWR models showing particularly promising outcomes. Nevertheless, higher prevalence rates persisted in Northern India and certain parts of the eastern region, most notably in Odisha (**Figure 5**).

4. Discussion

The study is the first attempt to measure preterm birth at the Indian district level by exploring the calendar data of NFHS [62]. The analysis establishes the evidence of the association between in-utero exposure to $PM_{2.5}$ and adverse birth outcomes by leveraging satellite data and large-scale survey data. The individual-level analysis reveals that an increase in ambient $PM_{2.5}$ is associated with a greater likelihood of LBW and PTB, consistent with previous studies [36,67–69]. We observe a higher value of the odds ratio of having preterm infants whose mothers were exposed to $PM_{2.5}$ during pregnancy as compared to the results for LBW. A significant role of indoor air pollution has been observed in the study for LBW. Further, climatic factors such as rainfall and temperature are significantly associated with ABOs in India. To the best of our knowledge, this study is the first of its kind to understand the association between in-utero exposure to $PM_{2.5}$ and adverse birth outcomes in India.

It is worth noting that except for Nagaland, North-Eastern states had a lower prevalence of preterm birth and low birth weight, coinciding with lower pollution levels in that region. Previous studies have found that the concentration of ambient $PM_{2.5}$ is highest in the states of upper-Gangetic plains such as Delhi, Punjab, Haryana, Uttar Pradesh and Bihar, as observed in the present study [70–73]. A recent Lancet study suggests that the average $PM_{2.5}$ concentrations in Delhi were 13.8 times higher than that in Kerala [74]. The Northern states had the highest $PM_{2.5}$ concentrations. The NFHS-5 report suggests that a large proportion of households in the Northern parts of India use solid fuels compared to other regions [46]. It is well documented that the residential sector is a significant contributor to the total $PM_{2.5}$ emissions along with the industry, energy and agriculture sectors [75]. Among industrial, residential and energy sources, the contribution of energy sources to total emissions is the maximum, while residential sources contribute the maximum to $PM_{2.5}$ emissions during winter and post-monsoon [75]. In contrast, certain studies conducted on future emissions scenarios in India forecast an increase in $PM_{2.5}$ levels [76]. However, at the urban or city level, where most households are already using cleaner fuel, reducing vehicular emissions (both exhaust and non-exhaust) emerges as a crucial strategy for reducing $PM_{2.5}$ levels. It was prominently observed during the coronavirus pandemic (COVID-19) lockdown in Indian cities when traffic reduction substantially minimized urban areas' exposure to air pollutants [70,77]. Moreover, the issue of air pollution is exacerbated by crop residue burning and forest fires in northern India, significantly contributing to the toxic air quality in the region [78,79]. The higher prevalence of low birth weight and preterm birth in the districts of the Northern region indicates a spatial association between $PM_{2.5}$ and birth outcomes. In line with our hypothesis, the study finds an individual level and spatial association between in-utero exposure to $PM_{2.5}$ and LBW and PTB.

The mechanisms behind preterm birth due to exposure to $PM_{2.5}$ are not clearly understood. Some epidemiological and toxicological studies proposed different pathways to explain the paradox. Due to the finer size of $PM_{2.5}$, inhaling particulate matter deposits in the lungs and affects the circulatory system [80]. Which is a reason of having oxidative stress, blood coagulation and placental inflammation that restricts the fetal growth [81–85]. Nevertheless, the presence of particulate matter in the human body disturbs oxygen transport and causes hormone dysfunction, which is the reason for placental insufficiency [86–89].

An earlier study identified that maternal exposure to $PM_{2.5}$ in the latter stages of pregnancy induces cytokine activation, favouring inflammation [90]. Sometimes inhaled $PM_{2.5}$ penetrates the toxic gases that damage deoxyribonucleic acid (DNA), restricting nutrient supply to the fetus resulting in pregnancy complications

during the last trimester of pregnancy that triggers the possibility of having a preterm birth [91,92]. Moreover, substantial exposure to a large amount of $PM_{2.5}$ has been linked to fetal malformation, miscarriage and stillbirth, all of which can influence subsequent birth outcomes [93–95].

Medical studies found that the mother's fetus grows rapidly in the third trimester of pregnancy [96,97]. That period is more sensitive, and exposure to $PM_{2.5}$ can hemorrhage the foetal growth easily [98,99]. Nevertheless, the level of thyroid hormone might be affected by the toxicity of $PM_{2.5}$, which is a responsible factor for less fetus weight [100]. Previous studies have found that fetal growth depends on many factors, such as mothers' health, socioeconomic condition and genetic factors [101,102,102,103]. Thus, the present study has adjusted for those potential plausible factors. Nevertheless, the study establishes a significant association between $PM_{2.5}$ and LBW. Further investigation is required to explore the biological mechanism by considering the biomarker measurement of the fetus.

Low birth weight and preterm birth significantly contribute to the highest number of stunting and premature deaths in India [104–106]. Since 1970, the Indian government has been trying to improve child health by adopting several schemes that promote the utilization of maternal healthcare facilities, spread awareness of reproductive health, supply nutrients to mothers and newborns, etc. Adverse birth outcomes not only impact a child's health but also reduce the productivity of the human resources of a country.

Understanding the impact of exposure to indoor air pollution during pregnancy is also important because pregnant women tend to spend most of their time indoors and this time only increases once the pregnancy progresses. Past studies found that releasing pollutants from uncleaned biomass burning in the household restricts fetal growth, increasing the probability of having a child with low weight and preterm birth [107,108]. In line with the hypothesis, our study also found a negative association between in-utero exposure to $PM_{2.5}$ and birth weight, whereas the result for PTB was the opposite. As PTB mostly depends on the mother’s health and obstetric factors [62], which could explain the absence of a discernible effect of $PM_{2.5}$ on PTB in the study. However, addressing indoor air pollution through interventions such as promoting cleaner fuels and improving home ventilation is crucial for improving pregnancy outcomes and neonatal health.

The present finds a significant role of climatic factors for ABOs. Previous evidence suggests that high temperatures can cause heat stress, dehydration, and reduced uteroplacental blood flow, impairing fetal growth and increasing the risk of LBW [109]. Additionally, heat-induced oxidative stress and inflammation can disrupt placental function, while cardiovascular strain and hormonal imbalances can further complicate pregnancy outcomes. On the other hand, extreme rainfall can heighten the risk of waterborne and vector-borne diseases, leading to maternal infections that adversely affect fetal development (Poursafa et al., 2015). Flooding can also result in nutritional deficiencies, physical and psychological stress, and disrupted healthcare access, all of which contribute to LBW and PTB [110,111]. Improved healthcare access, infrastructure development, and policies addressing climate change are essential to protect maternal and neonatal health against these challenges.

Our findings suggest that both indoor and ambient air pollution along with climatic factor are significantly associated with ABOs. However, to gain a deeper understanding of the biological mechanisms underpinning the relationship between air pollution and malnutrition, which cannot be fully explored using existing datasets, conducting an in-depth epidemiological study is imperative. It is important to note that our research assumes

that mothers did not change their residence during the period from pregnancy to the conducting date of the survey. The study acknowledges that like some other possible determinants, smoking practices and alcohol consumption were not considered in the model due to the under-reporting nature. Also, the possibility of biases due to self-reporting cannot be ignored. Due to the cross-sectional nature of the study, we cannot draw a causal relationship, other aspects including the seasonality effects can be explored using the longitudinal study in the future. As India is not satisfactorily progressing in child nutrition, hence consorted effort to improve environmental conditions, especially air quality, is essential to tackle issues related to child health.

5. Conclusion

The present study provides robust evidence linking in-utero exposure to ambient $PM_{2.5}$ and climatic factors, such as temperature and rainfall, with adverse birth outcomes in India. The geostatistical analysis underscores the need for targeted interventions, particularly in Northern districts identified as highly vulnerable. To address these challenges, comprehensive strategies are essential. The National Clean Air Program should be intensified, with stricter emission standards and enhanced air quality monitoring. Integrating air quality data with health surveillance systems will enable precise identification of at-risk populations. Additionally, policies promoting clean cooking fuels and energy-efficient technologies can reduce indoor air pollution. Climate adaptation strategies, such as developing heat action plans and improving water management, should be incorporated into public health planning to mitigate the effects of extreme temperatures and irregular rainfall. Public health initiatives must raise awareness about the risks of air pollution and climate change, particularly among pregnant women.

Statements & Declarations

Funding

No financial assistance has been granted from any survey organization, institutional open access or any other alternative sources to carry out the research.

Data Availability

The study uses secondary data that are available on reasonable request through https://dhsprogram.com/data/dataset_admin/

Ethics approval

The Indian Demographic and Health Survey (DHS) is known as National Family Health Survey (NFHS) in India. We used published large scale national data where every respondent was anonymized in the data set itself. As it is not based on a primary survey- cases, we need not to do any anonymization in the study as the data is already made in that fashion following all ethical protocols. Thereby, it is certified that all applicable institutional and governmental regulations concerning the ethical use of human volunteers were followed during the course of the survey.

Consent to participate

Verbal as well as written informed consent was obtained from all the participants. The informed consent was taken from their parent or legal guardian who were not mature or below 18 aged. Then blood sample was taken from the finger and collected in microcuvette. The data is in the public domain- free of cost for users and funded by the government. More details on that available at https://dhsprogram.com/search/index.cfm?bydoctype=publication&bypubtype=5.

Consent to publish

The dataset is publicly available, thus consent for publication is not applicable for the study.

References

1. Monteiro A, Sá E, Fernandes A, et al. How healthy will be the air quality in 2050? *Air Qual Atmos Health*. 2018;11(3):353-362. doi:10.1007/s11869-017-0466-z

2. UN. COP26: Together for our planet. Published online 2021. Accessed October 13, 2022. https://www.un.org/en/climatechange/cop26

3. Jana A, Varghese JS, Naik G. Household air pollution and cognitive health among Indian older adults: Evidence from LASI. *Environ Res*. 2022;214(Pt 1):113880. doi:10.1016/j.envres.2022.113880

4. Murray CJL, Aravkin AY, Zheng P, et al. Global burden of 87 risk factors in 204 countries and territories, 1990–2019: a systematic analysis for the Global Burden of Disease Study 2019. *The Lancet*. 2020;396(10258):1223-1249. doi:10.1016/S0140-6736(20)30752-2

5. World Bank, Evaluation I for HM and. *The Cost of Air Pollution: Strengthening the Economic Case for Action*. World Bank; 2016. doi:10.1596/25013

6. Korhonen A, Lehtomäki H, Rumrich I, et al. Influence of spatial resolution on population PM2.5 exposure and health impacts. *Air Qual Atmos Health*. 2019;12(6):705-718. doi:10.1007/s11869-019-00690-z

7. IHME. GBD 2019 Cause and Risk Summary: Ambient particulate matter pollution. Published online 2020.

8. Bachwenkizi J, Liu C, Meng X, et al. Fine particulate matter constituents and infant mortality in Africa: A multicountry study. *Environment International*. 2021;156:106739. doi:10.1016/j.envint.2021.106739

9. Usmani RSA, Saeed A, Abdullahi AM, Pillai TR, Jhanjhi NZ, Hashem IAT. Air pollution and its health impacts in Malaysia: a review. *Air Qual Atmos Health*. 2020;13(9):1093-1118. doi:10.1007/s11869-020-00867-x

10. Fann N, Risley D. The public health context for PM2.5 and ozone air quality trends. *Air Qual Atmos Health*. 2013;6(1):1-11. doi:10.1007/s11869-010-0125-0

11. WHO. Ageing and health. Published 2022. Accessed July 17, 2024. https://www.who.int/news-room/fact-sheets/detail/ageing-and-health

12. Abed Al Ahad M, Demšar U, Sullivan F, Kulu H. Long-term exposure to air pollution and mortality in Scotland: A register-based individual-level longitudinal study. *Environmental Research*. 2023;238:117223. doi:10.1016/j.envres.2023.117223

13. Anderson HR, Favarato G, Atkinson RW. Long-term exposure to air pollution and the incidence of asthma: meta-analysis of cohort studies. *Air Qual Atmos Health*. 2013;6(1):47-56. doi:10.1007/s11869-011-0144-5

14. WHO. Factsheet: Ambient (outdoor) air pollution. Published online 2021. Accessed October 13, 2022. https://www.who.int/news-room/fact-sheets/detail/ambient-(outdoor)-air-quality-and-health

15. WHO. 7 million premature deaths annually linked to air pollution. Published online 2014. https://www.who.int/news/item/25-03-2014-7-million-premature-deaths-annually-linked-to-air-pollution

16. World Bank. *Getting Down to Earth: Are Satellites Reliable for Measuring Air Pollutants That Cause Mortality in Low- and Middle-Income Countries?* World Bank; 2022. doi:10.1596/978-1-4648-1727-4

17. IQAir. World Air Quality Report 2023. Published online 2023.

18. Yadav R, Deora S, Yadav G. Air pollution and its impact on cardiovascular health – It's time to act fast! *Indian Heart J*. 2021;73(1):1-6. doi:10.1016/j.ihj.2021.01.021

19. Pandey A, Brauer M, Cropper ML, et al. Health and economic impact of air pollution in the states of India: the Global Burden of Disease Study 2019. *The Lancet Planetary Health*. 2021;5(1):e25-e38. doi:10.1016/S2542-5196(20)30298-9

20. Chen B, Kan H. Air pollution and population health: a global challenge. *Environ Health Prev Med*. 2008;13(2):94-101. doi:10.1007/s12199-007-0018-5

21. Samet JM, Chung YS. Air quality, atmosphere, and health. *Air Qual Atmos Health*. 2008;1(1):1-2. doi:10.1007/s11869-008-0007-x

22. EPIC. Air Quality Life Index: India Fact sheet. Published online 2022. Accessed October 13, 2022. https://aqli.epic.uchicago.edu/wp-content/uploads/2022/06/IndiaFactSheet_2022_India-version.pdf

23. World Bank. Getting Down to Earth: Are Satellites Reliable for Measuring Air Pollutants that Cause Mortality in Low- and Middle-Income Countries? International Development in Focus. Published online 2021.

24. Brumberg HL, Karr CJ, COUNCIL ON ENVIRONMENTAL HEALTH. Ambient Air Pollution: Health Hazards to Children. *Pediatrics*. 2021;147(6):e2021051484. doi:10.1542/peds.2021-051484

25. Mahapatra B, Walia M, Avis WR, Saggurti N. Effect of exposure to PM10 on child health: evidence based on a large-scale survey from 184 cities in India. *BMJ Glob Health*. 2020;5(8):e002597. doi:10.1136/bmjgh-2020-002597

26. Guarnieri M, Balmes JR. Outdoor air pollution and asthma. *Lancet*. 2014;383(9928):1581-1592. doi:10.1016/S0140-6736(14)60617-6

27. Stieb DM, Chen L, Eshoul M, Judek S. Ambient air pollution, birth weight and preterm birth: a systematic review and meta-analysis. *Environ Res*. 2012;117:100-111. doi:10.1016/j.envres.2012.05.007

28. Solomon PA, Costantini M, Grahame TJ, et al. Air pollution and health: bridging the gap from sources to health outcomes: conference summary. *Air Qual Atmos Health*. 2012;5(1):9-62. doi:10.1007/s11869-011-0161-4

29. Kumar P, Kausar MohdA, Singh AB, Singh R. Biological contaminants in the indoor air environment and their impacts on human health. *Air Qual Atmos Health*. 2021;14(11):1723-1736. doi:10.1007/s11869-021-00978-z

30. Orru H, Maasikmets M, Lai T, et al. Health impacts of particulate matter in five major Estonian towns: main sources of exposure and local differences. *Air Qual Atmos Health*. 2011;4(3):247-258. doi:10.1007/s11869-010-0075-6

31. Dadvand P, Parker J, Bell ML, et al. Maternal exposure to particulate air pollution and term birth weight: a multi-country evaluation of effect and heterogeneity. *Environ Health Perspect*. 2013;121(3):267-373. doi:10.1289/ehp.1205575

32. Shah PS, Balkhair T, Knowledge Synthesis Group on Determinants of Preterm/LBW births. Air pollution and birth outcomes: a systematic review. *Environ Int*. 2011;37(2):498-516. doi:10.1016/j.envint.2010.10.009

33. Risnes KR, Vatten LJ, Baker JL, et al. Birthweight and mortality in adulthood: a systematic review and meta-analysis. *Int J Epidemiol*. 2011;40(3):647-661. doi:10.1093/ije/dyq267

34. WHO. Factsheet: Preterm birth. Published online 2018. Accessed October 13, 2022. https://www.who.int/news-room/fact-sheets/detail/preterm-birth

35. UNICEF. Low birth weight. Published online 2022. https://data.unicef.org/topic/nutrition/low-birthweight/

36. Goyal N, Canning D. The association of in-utero exposure to ambient fine particulate air pollution with low birth weight in India. *Environ Res Lett*. 2021;16(5):054034. doi:10.1088/1748-9326/abf18e

37. Shah PS, Balkhair T. Air pollution and birth outcomes: A systematic review. *Environment International*. 2011;37(2):498-516. doi:10.1016/j.envint.2010.10.009

38. Balakrishnan K, Ghosh S, Thangavel G, et al. Exposures to fine particulate matter (PM2.5) and birthweight in a rural-urban, mother-child cohort in Tamil Nadu, India. *Environ Res*. 2018;161:524-531. doi:10.1016/j.envres.2017.11.050

39. Balakrishnan K, Ghosh S, Thangavel G, et al. Exposures to fine particulate matter (PM2.5) and birthweight in a rural-urban, mother-child cohort in Tamil Nadu, India. *Environ Res*. 2018;161:524-531. doi:10.1016/j.envres.2017.11.050

40. Bachwenkizi J, Liu C, Meng X, et al. Maternal exposure to fine particulate matter and preterm birth and low birth weight in Africa. *Environ Int*. 2022;160:107053. doi:10.1016/j.envint.2021.107053

41. Huang FH. Explore Home Care Needs and Satisfaction for Elderly People with Chronic Disease and their Family Members. *Procedia Manufacturing*. 2015;3:173-179. doi:10.1016/j.promfg.2015.07.123

42. Li Q, Wang Y yuan, Guo Y, et al. Effect of airborne particulate matter of 2.5 μm or less on preterm birth: A national birth cohort study in China. *Environment International*. 2018;121:1128-1136. doi:10.1016/j.envint.2018.10.025

43. Liu X, Zhu Y, Xue L, Desai AR, Wang H. Cluster-Enhanced Ensemble Learning for Mapping Global Monthly Surface Ozone From 2003 to 2019. *Geophysical Research Letters*. 2022;49(11):e2022GL097947. doi:10.1029/2022GL097947

44. Chawanpaiboon S, Vogel JP, Moller AB, et al. Global, regional, and national estimates of levels of preterm birth in 2014: a systematic review and modelling analysis. *The Lancet Global Health*. 2019;7(1):e37-e46. doi:10.1016/S2214-109X(18)30451-0

45. UNDESA. 2018 Revision of World Urbanization Prospects. Published online 2018. https://www.un.org/development/desa/publications/2018-revision-of-world-urbanization-prospects.html

46. IIPS, ICF. National Family Healh Survey. *international Institute for Population Sciences, Mumbai*. Published online 2022. http://rchiips.org/nfhs/NFHS-5Reports/NFHS-5_INDIA_REPORT.pdf

47. van Donkelaar A, Hammer MS, Bindle L, et al. Monthly Global Estimates of Fine Particulate Matter and Their Uncertainty. *Environ Sci Technol*. 2021;55(22):15287-15300. doi:10.1021/acs.est.1c05309

48. Boing AF, deSouza P, Boing AC, Kim R, Subramanian SV. Air Pollution, Socioeconomic Status, and Age-Specific Mortality Risk in the United States. *JAMA Network Open*. 2022;5(5):e2213540. doi:10.1001/jamanetworkopen.2022.13540

49. Yu Z, Hu N, Du Y, et al. Association of outdoor artificial light at night with mental health among China adults: a prospective ecology study. *Environ Sci Pollut Res*. Published online June 25, 2022. doi:10.1007/s11356-022-21587-y

50. Ran Q, Lee SY, Zheng D, et al. Potential health and economic impacts of shifting manufacturing from China to Indonesia or India. *Science of The Total Environment*. 2023;855:158634. doi:10.1016/j.scitotenv.2022.158634

51. Jin H, Zhong R, Liu M, Ye C, Chen X. Spatiotemporal distribution characteristics of PM2.5 concentration in China from 2000 to 2018 and its impact on population. *Journal of Environmental Management*. 2022;323:116273. doi:10.1016/j.jenvman.2022.116273

52. de Leeuw G, Fan C, Li Z, et al. Spatiotemporal variation and provincial scale differences of the AOD across China during 2000–2021. *Atmospheric Pollution Research*. 2022;13(4):101359. doi:10.1016/j.apr.2022.101359

53. Wensu Z, Wen C, Fenfen Z, Wenjuan W, Li L. The Association Between Long-Term Exposure to Particulate Matter and Incidence of Hypertension Among Chinese Elderly: A Retrospective Cohort Study. *Front Cardiovasc Med*. 2022;8:784800. doi:10.3389/fcvm.2021.784800

54. Zhang L, Wilson JP, Zhao N, Zhang W, Wu Y. The dynamics of cardiovascular and respiratory deaths attributed to long-term PM2.5 exposures in global megacities. *Science of The Total Environment*. 2022;842:156951. doi:10.1016/j.scitotenv.2022.156951

55. Peng J, Zheng Y, Liu C. The Impact of Urban Construction Land Use Change on Carbon Emissions: Evidence from the China Land Market in 2000–2019. *Land*. 2022;11(9):1440. doi:10.3390/land11091440

56. Shen Z, Yong B, Gourley JJ, et al. Recent global performance of the Climate Hazards group Infrared Precipitation (CHIRP) with Stations (CHIRPS). *Journal of Hydrology*. 2020;591:125284. doi:10.1016/j.jhydrol.2020.125284

57. Muñoz-Sabater J, Dutra E, Agustí-Panareda A, et al. ERA5-Land: a state-of-the-art global reanalysis dataset for land applications. *Earth System Science Data*. 2021;13(9):4349-4383. doi:10.5194/essd-13-4349-2021

58. Peng J, Dadson S, Hirpa F, et al. A pan-African high-resolution drought index dataset. *Earth System Science Data*. 2020;12(1):753-769. doi:10.5194/essd-12-753-2020

59. Jana A, Chattopadhyay A. Drought and Socioeconomic Drivers of Crop Diversity in India: A Panel Analysis. *Agric Res*. Published online August 7, 2023. doi:10.1007/s40003-023-00665-8

60. Dinku T, Funk C, Peterson P, et al. Validation of the CHIRPS satellite rainfall estimates over eastern Africa. *Quarterly Journal of the Royal Meteorological Society*. 2018;144(S1):292-312. doi:10.1002/qj.3244

61. WHO. Preterm birth. Geneva: WHO, Updated Nov 2015. Published online 2015. Accessed August 20, 2021. http://www.who.int/mediacentre/ factsheets/fs363/en/.

62. Jana A, Banerjee K, Khan PK. Early arrivals: association of maternal obstetric factors with preterm births and their survival in India. *Public Health*. 2022;211:37-46. doi:10.1016/j.puhe.2022.07.005

63. Gathimba NW, Wanjoya A, Kiplagat GK, Mbugua L, Kibiwott K. Modeling Maternal Risk Factors Affecting Low Birth Weight Among Infants in Kenya. *American Journal of Theoretical and Applied Statistics*. 2017;6(1):22. doi:10.11648/j.ajtas.20170601.13

64. Tellapragada C, Eshwara VK, Bhat P, et al. Risk Factors for Preterm Birth and Low Birth Weight Among Pregnant Indian Women: A Hospital-based Prospective Study. *J Prev Med Public Health*. 2016;49(3):165-175. doi:10.3961/jpmph.16.022

65. Fotheringham AS, Yang W, Kang W. Multiscale Geographically Weighted Regression (MGWR). *Annals of the American Association of Geographers*. 2017;107(6):1247-1265. doi:10.1080/24694452.2017.1352480

66. Lam C, Souza PCL. Estimation and Selection of Spatial Weight Matrix in a Spatial Lag Model. *Journal of Business & Economic Statistics*. 2020;38(3):693-710. doi:10.1080/07350015.2019.1569526

67. Balakrishnan K, Ghosh S, Thangavel G, et al. Exposures to fine particulate matter (PM2.5) and birthweight in a rural-urban, mother-child cohort in Tamil Nadu, India. *Environmental Research*. 2018;161:524-531. doi:10.1016/j.envres.2017.11.050

68. Fleischer NL, Merialdi M, van DA, et al. Outdoor Air Pollution, Preterm Birth, and Low Birth Weight: Analysis of the World Health Organization Global Survey on Maternal and Perinatal Health. *Environmental Health Perspectives*. 2014;122(4):425-430. doi:10.1289/ehp.1306837

69. Sun X, Luo X, Zhao C, et al. The associations between birth weight and exposure to fine particulate matter (PM2.5) and its chemical constituents during pregnancy: A meta-analysis. *Environ Pollut*. 2016;211:38-47. doi:10.1016/j.envpol.2015.12.022

70. Das M, Das A, Sarkar R, Saha S, Mandal A. Examining the impact of lockdown (due to COVID-19) on ambient aerosols (PM2.5): A study on Indo-Gangetic Plain (IGP) Cities, India. *Stoch Environ Res Risk Assess*. 2021;35(6):1301-1317. doi:10.1007/s00477-020-01905-x

71. Jat R, Gurjar BR. Contribution of different source sectors and source regions of Indo-Gangetic Plain in India to PM2.5 pollution and its short-term health impacts during peak polluted winter. *Atmospheric Pollution Research*. 2021;12(4):89-100. doi:10.1016/j.apr.2021.02.016

72. Mhawish A, Banerjee T, Sorek-Hamer M, et al. Estimation of High-Resolution PM2.5 over the Indo-Gangetic Plain by Fusion of Satellite Data, Meteorology, and Land Use Variables. *Environ Sci Technol*. 2020;54(13):7891-7900. doi:10.1021/acs.est.0c01769

73. Singh N, Murari V, Kumar M, Barman SC, Banerjee T. Fine particulates over South Asia: Review and meta-analysis of PM2.5 source apportionment through receptor model. *Environmental Pollution*. 2017;223:121-136. doi:10.1016/j.envpol.2016.12.071

74. Pandey A, Brauer M, Cropper ML, et al. Health and economic impact of air pollution in the states of India: the Global Burden of Disease Study 2019. *The Lancet Planetary Health*. 2021;5(1):e25-e38. doi:10.1016/S2542-5196(20)30298-9

75. Guo H, Kota SH, Sahu SK, et al. Source apportionment of PM2.5 in North India using source-oriented air quality models. *Environmental Pollution*. 2017;231:426-436. doi:10.1016/j.envpol.2017.08.016

76. Venkataraman C, Brauer M, Tibrewal K, et al. Source influence on emission pathways and ambient $PM_{2.5}$ pollution over India (2015–2050). *Atmospheric Chemistry and Physics*. 2018;18(11):8017-8039. doi:10.5194/acp-18-8017-2018

77. Singh V, Singh S, Biswal A, Kesarkar AP, Mor S, Ravindra K. Diurnal and temporal changes in air pollution during COVID-19 strict lockdown over different regions of India. *Environmental Pollution*. 2020;266:115368. doi:10.1016/j.envpol.2020.115368

78. Saxena P, Sonwani S, Srivastava A, et al. Impact of crop residue burning in Haryana on the air quality of Delhi, India. *Heliyon*. 2021;7(5):e06973. doi:10.1016/j.heliyon.2021.e06973

79. Singh T, Biswal A, Mor S, Ravindra K, Singh V, Mor S. A high-resolution emission inventory of air pollutants from primary crop residue burning over Northern India based on VIIRS thermal anomalies. *Environmental Pollution*. 2020;266:115132. doi:10.1016/j.envpol.2020.115132

80. Xing YF, Xu YH, Shi MH, Lian YX. The impact of PM2.5 on the human respiratory system. *J Thorac Dis*. 2016;8(1):E69-E74. doi:10.3978/j.issn.2072-1439.2016.01.19

81. Deng X, Zhang F, Rui W, et al. PM2.5-induced oxidative stress triggers autophagy in human lung epithelial A549 cells. *Toxicol In Vitro*. 2013;27(6):1762-1770. doi:10.1016/j.tiv.2013.05.004

82. Fongsodsri K, Chamnanchanunt S, Desakorn V, et al. Particulate Matter 2.5 and Hematological Disorders From Dust to Diseases: A Systematic Review of Available Evidence. *Frontiers in Medicine*. 2021;8. Accessed October 17, 2022. https://www.frontiersin.org/articles/10.3389/fmed.2021.692008

83. Kaur K, Lesseur C, Deyssenroth MA, et al. PM2.5 exposure during pregnancy is associated with altered placental expression of lipid metabolic genes in a US birth cohort. *Environmental Research*. 2022;211:113066. doi:10.1016/j.envres.2022.113066

84. Leung M, Weisskopf MG, Laden F, et al. Exposure to PM2.5 during Pregnancy and Fetal Growth in Eastern Massachusetts, USA. *Environmental Health Perspectives*. 130(1):017004. doi:10.1289/EHP9824

85. Liu Y, Wang L, Wang F, Li C. Effect of Fine Particulate Matter (PM2.5) on Rat Placenta Pathology and Perinatal Outcomes. *Med Sci Monit*. 2016;22:3274-3280. doi:10.12659/MSM.897808

86. Li H, Cai J, Chen R, et al. Particulate Matter Exposure and Stress Hormone Levels. *Circulation*. 2017;136(7):618-627. doi:10.1161/CIRCULATIONAHA.116.026796

87. Sonwani S, Madaan S, Arora J, et al. Inhalation Exposure to Atmospheric Nanoparticles and Its Associated Impacts on Human Health: A Review. *Frontiers in Sustainable Cities*. 2021;3. Accessed October 17, 2022. https://www.frontiersin.org/articles/10.3389/frsc.2021.690444

88. Zhou S, Xi Y, Chen Y, et al. Ovarian Dysfunction Induced by Chronic Whole-Body PM2.5 Exposure. *Small*. 2020;16(33):2000845. doi:10.1002/smll.202000845

89. Zhu N, Ji X, Geng X, Yue H, Li G, Sang N. Maternal PM2.5 exposure and abnormal placental nutrient transport. *Ecotoxicology and Environmental Safety*. 2021;207:111281. doi:10.1016/j.ecoenv.2020.111281

90. Nachman RM, Mao G, Zhang X, et al. Intrauterine Inflammation and Maternal Exposure to Ambient PM2.5 during Preconception and Specific Periods of Pregnancy: The Boston Birth Cohort. *Environ Health Perspect*. 2016;124(10):1608-1615. doi:10.1289/EHP243

91. Alman BL, Stingone JA, Yazdy M, et al. Associations between PM2.5 and risk of preterm birth among liveborn infants. *Ann Epidemiol*. 2019;39:46-53.e2. doi:10.1016/j.annepidem.2019.09.008

92. Zhang X, Fan C, Ren Z, et al. Maternal PM2.5 exposure triggers preterm birth: a cross-sectional study in Wuhan, China. *Global Health Research and Policy*. 2020;5(1):17. doi:10.1186/s41256-020-00144-5

93. Grippo A, Zhang J, Chu L, et al. Air pollution exposure during pregnancy and spontaneous abortion and stillbirth. *Rev Environ Health*. 2018;33(3):247-264. doi:10.1515/reveh-2017-0033

94. Wilcox AJ, Morken NH, Weinberg CR, Håberg SE, Magnus MC. Role of maternal age and pregnancy history in risk of miscarriage: prospective register based study. *BMJ*. 2019;364:l869. doi:10.1136/bmj.l869

95. Xie G, Sun L, Yang W, et al. Maternal exposure to PM2.5 was linked to elevated risk of stillbirth. *Chemosphere*. 2021;283:131169. doi:10.1016/j.chemosphere.2021.131169

96. Deng Q, Lu C, Li Y, Sundell J, Dan Norbäck null. Exposure to outdoor air pollution during trimesters of pregnancy and childhood asthma, allergic rhinitis, and eczema. *Environ Res*. 2016;150:119-127. doi:10.1016/j.envres.2016.05.050

97. Langford K, Nicolaides K, Miell JP. Maternal and fetal insulin-like growth factors and their binding proteins in the second and third trimesters of human pregnancy. *Human Reproduction*. 1998;13(5):1389-1393. doi:10.1093/humrep/13.5.1389

98. Fleisch AF, Rifas-Shiman SL, Koutrakis P, et al. Prenatal Exposure to Traffic Pollution: Associations with Reduced Fetal Growth and Rapid Infant Weight Gain. *Epidemiology*. 2015;26(1):43-50. doi:10.1097/EDE.0000000000000203

99. Karachaliou M, Georgiou V, Roumeliotaki T, et al. Association of trimester-specific gestational weight gain with fetal growth, offspring obesity, and cardiometabolic traits in early childhood. *American Journal of Obstetrics and Gynecology*. 2015;212(4):502.e1-502.e14. doi:10.1016/j.ajog.2014.12.038

100. Janssen BG, Saenen ND, Roels HA, et al. Fetal Thyroid Function, Birth Weight, and in Utero Exposure to Fine Particle Air Pollution: A Birth Cohort Study. *Environ Health Perspect*. 2017;125(4):699-705. doi:10.1289/EHP508

101. Roland MCP, Friis CM, Godang K, Bollerslev J, Haugen G, Henriksen T. Maternal Factors Associated with Fetal Growth and Birthweight Are Independent Determinants of Placental Weight and Exhibit Differential Effects by Fetal Sex. *PLoS One*. 2014;9(2):e87303. doi:10.1371/journal.pone.0087303

102. Vorherr H. Factors influencing fetal growth. *Am J Obstet Gynecol*. 1982;142(5):577-588. doi:10.1016/0002-9378(82)90765-7

103. Workalemahu T, Grantz KL, Grewal J, Zhang C, Louis GMB, Tekola-Ayele F. Genetic and Environmental Influences on Fetal Growth Vary during Sensitive Periods in Pregnancy. *Sci Rep*. 2018;8(1):7274. doi:10.1038/s41598-018-25706-z

104. Chawla D. Survival of Very-Low-Birth-Weight Neonates in India. *Indian J Pediatr*. 2021;88(4):326-327. doi:10.1007/s12098-021-03710-y

105. Halli SS, Biradar RA, Prasad JB. Low Birth Weight, the Differentiating Risk Factor for Stunting among Preschool Children in India. *International Journal of Environmental Research and Public Health*. 2022;19(7):3751. doi:10.3390/ijerph19073751

106. Swaminathan S, Hemalatha R, Pandey A, et al. The burden of child and maternal malnutrition and trends in its indicators in the states of India: the Global Burden of Disease Study 1990–2017. *The Lancet Child & Adolescent Health*. 2019;3(12):855-870. doi:10.1016/S2352-4642(19)30273-1

107. Epstein MB, Bates MN, Arora NK, Balakrishnan K, Jack DW, Smith KR. Household fuels, low birth weight, and neonatal death in India: the separate impacts of biomass, kerosene, and coal. *Int J Hyg Environ Health*. 2013;216(5):523-532. doi:10.1016/j.ijheh.2012.12.006

108. Islam S, Mohanty SK. Maternal exposure to cooking smoke and risk of low birth weight in India. *Science of The Total Environment*. 2021;774:145717. doi:10.1016/j.scitotenv.2021.145717

109. Bonell A, Sonko B, Badjie J, et al. Environmental heat stress on maternal physiology and fetal blood flow in pregnant subsistence farmers in The Gambia, west Africa: an observational cohort study. *The Lancet Planetary Health*. 2022;6(12):e968-e976. doi:10.1016/S2542-5196(22)00242-X

110. Hunter PR. Climate change and waterborne and vector-borne disease. *J Appl Microbiol*. 2003;94 Suppl:37S-46S. doi:10.1046/j.1365-2672.94.s1.5.x

111. Rodriguez-Llanes JM, Ranjan-Dash S, Mukhopadhyay A, Guha-Sapir D. Flood-Exposure is Associated with Higher Prevalence of Child Undernutrition in Rural Eastern India. *International Journal of Environmental Research and Public Health*. 2016;13(2):210. doi:10.3390/ijerph13020210

Table 1. Sample distribution used in the analysis

Variables	Sample	Percentage
**Preterm birth**		
No	2,01,657	87.45
Yes	28,933	12.55
**Low birth weight**		
No	1,71,116	82.6
Yes	36,039	17.4
**Sex of the child**		
Male	1,19,474	51.81
Female	1,11,116	48.19
**Birth order**		
1	88,235	38.26
2	1,11,898	48.53
3+	30,457	13.21
**Mother's age at delivery**		
Below 20	26,282	11.4
20-24	98,137	42.56
25-29	68,494	29.7
30&above	37,677	16.34
**Place of delivery**		
Home	31,331	13.59
Hospital	1,99,259	86.41
**Place of residence**		
Rural	1,84,038	79.81
Urban	46,552	20.19
**Mother's body mass index**		
Underweight	42,112	18.73
Normal	1,42,504	63.38
Overweight/obese	40,208	17.88
**Mother's education**		
Illiterate/primary	80,636	34.97
Secondary	1,18,602	51.43
Higher	31,352	13.6
**Wealth status**		
Poor	1,16,744	50.63
Middle	44,700	19.39
Rich	69,146	29.99
**Religion**		
Hindu	1,69,233	73.39
Muslim	33,194	14.4
Others	28,163	12.21
**Cooking fuel**		
Clean fuel	97,582	42.32
Solid fuel	1,33,008	57.68

Table 2. Adjusted and unadjusted odds ratio showing the effects of in-utero exposure to $PM_{2.5}$ on low birth weight and preterm birth

Determinants	Low birth weight		Preterm birth	
	OR	AOR	OR	AOR
**$PM_{2.5}$ ($\mu g/m^3$)**				
**< mean**				
**>= mean**	1.56***(1.50 1.63)	1.37***(1.29 1.45)	1.62***(1.55 1.69)	1.67***(1.57 1.77)
**Temperature (°C)**				
**< mean**				
**>= mean**		1.14***(1.01 1.04)		0.98 (0.96 1.01)
**Rainfall (mm)**				
**< mean**				
**>= mean**		1.07***(1.03 1.12)		1.04***(1.02 1.10)
**Sex of the child**				
Male (Ref)				
Female		1.18***(1.15 1.20)		0.97**(0.95 1)
**Birth order**				
1 (Ref)				
2		0.89***(0.86 0.91)		0.99 (0.96 1.02)
3+		0.84***(0.8 0.88)		0.94***(0.89 0.98)
**Mother's age at delivery**				
Below 20		1.09***(1.04 1.15)		1.08***(1.02 1.14)
20-24		1.03 (0.99 1.07)		1.03 (0.98 1.07)
25-29		1 (0.96 1.03)		0.97 (0.93 1.01)
30&above (Ref)				
**Place of delivery**				
Home (Ref)		1.11***(1.06 1.16)		1.00 (0.97 1.04)
Hospital				
**Place of residence**				
Rural (Ref)				
Urban		1.07***(1.04 1.11)		0.96**(0.92 0.99)
**Mother's body mass index**				
Normal (Ref)				
Underweight		1.27***(1.24 1.31)		1.04***(1.01 1.08)
Overweight/obese		0.93***(0.9 0.96)		0.95***(0.92 0.99)
**Mother's education**				
Illiterate/primary		1.40***(1.34 1.46)		
Secondary		1.23***(1.18 1.28)		1.00 (0.95 1.04)
Higher (Ref)				0.95**(0.91 0.99)
**Wealth status**				
Poor		1.09***(1.05 1.13)		0.92***(0.88 0.95)
Middle		0.98 (0.94 1.02)		0.95**(0.92 0.99)
Rich (Ref)				
**Religion**				
Hindu (Ref)				
Muslim		0.87***(0.84 0.91)		0.97 (0.94 1.01)
Others		0.70***(0.67 0.73)		0.88***(0.84 0.92)
**Cooking fuel**				
Clean fuel (Ref)				
Solid fuel		1.04 ***(1.01 1.07)		0.94 (0.91 1.03)

Note: Ref: Reference category; *** $p < 0.001$, ** < 0.05, * $p < 0.1$

Table 3. Sensitivity analysis of the effects of $PM_{2.5}$ on low birth weight and preterm birth

**Adjusted characteristic**	**Low birth weight**				**Preterm birth**			
	Odds ratio	P value	95% CI		Odds ratio	P value	95% CI	
			Lower	Upper			Lower	Upper
**Unadjusted**	1.56	<0.00	1.50	1.63	1.62	<0.00	1.55	1.69
Temperature (°C)	1.46	<0.00	1.40	1.52	1.69	<0.00	1.62	1.76
Climatic factors (temperature + rainfall)	1.39	<0.00	1.33	1.45	1.57	<0.00	1.50	1.65
Cooking fuel	1.53	<0.00	1.47	1.60	1.63	<0.00	1.57	1.71
Child characteristics	1.59	<0.00	1.51	1.68	1.76	<0.00	1.67	1.87
Mothers' characteristics	1.52	<0.00	1.45	1.58	1.61	<0.00	1.54	1.69
Socioeconomic factors	1.48	<0.00	1.42	1.54	1.59	<0.00	1.52	1.66
Socioeconomic, child and mothers' Characteristics	1.45	<0.00	1.37	1.54	1.70	<0.00	1.60	1.80
All the determinants	1.37	<0.00	1.29	1.45	1.67	<0.00	1.57	1.77

Table 4. spatial regression models showing the spatial association between $PM_{2.5}$ and low birth weight adjusted confounding factors

Variables	OLS		GWR		MGWR		SLM		SEM	
	Coefficient	SE	Coefficient	SE	Coefficient	SE	Coefficient	SE	Coefficient	SE
**$PM_{2.5}$** (µg/m^3)	0.059	0.009	0.122	0.009	0.119	0.016	0.043	0.014	0.031	0.008
Underweighted mother	0.159	0.031	0.179	0.007	0.163	0.003	0.124	0.032	0.102	0.027
Illiterate mother	0.061	0.018	0.095	0.009	0.013	0.001	0.068	0.021	0.040	0.016
Belong to poor family	-0.053	0.015	0.234	0.008	0.494	0.000	-0.011	0.019	-0.022	0.013
Hindu religion	0.010	0.009	-0.055	0.006	-0.017	0.002	0.007	0.011	-0.002	0.008
Non-institutional delivery	-0.083	0.025	-0.067	0.008	-0.155	0.000	-0.060	0.027	-0.057	0.022
Using solid cooking fuel	0.062	0.014	0.126	0.009	-0.118	0.000	0.024	0.017	0.028	0.013
Belong to rural areas	-0.018	0.012	-0.137	0.008	-0.114	0.005	0.002	0.012	-0.001	0.011
Female child	-0.005	0.058	0.003	0.003	0.001	0.004	0.008	0.048	0.001	0.050
Teen aged mothers	0.022	0.034	0.066	0.007	0.101	0.007	0.023	0.040	0.005	0.030
Having 3& more births	-0.090	0.046	-0.122	0.007	-0.03	0.005	-0.102	0.048	-0.069	0.040
Average rainfall (°C)	0.000	0.003	-0.071	0.005	-0.055	0.000	-0.003	0.003	0.001	0.003
Average temperature (mm)	0.041	0.041	0.026	0.007	0.165	0.000	0.105	0.052	0.023	0.036
R^2	0.34				0.68		0.49		0.52	
**AIC**	4192.36				1420.83		4045.78		4022.85	

Table 5. spatial regression models showing the spatial association between $PM_{2.5}$ and preterm birth adjusted confounding factors

**Variables**	**OLS**		**GWR**		**MGWR**		**SLM**		**SEM**	
	Coefficient	SE	Coefficient	SE	Coefficient	SE	Coefficient	SE	Coefficient	SE
**$PM_{2.5}$** ($\mu g/m^3$)	0.043	0.016	0.053	0.012	0.041	0.018	0.018	0.014	0.035	0.024
Underweighted mother	0.009	0.053	0.051	0.014	0.044	0.012	-0.022	0.046	-0.059	0.056
Illiterate mother	0.035	0.030	0.169	0.011	0.214	0.000	0.030	0.026	0.070	0.036
Belong to poor family	-0.055	0.026	-0.121	0.018	-0.140	0.000	-0.024	0.022	-0.029	0.033
Hindu religion	0.050	0.016	0.078	0.010	0.038	0.003	0.023	0.014	0.023	0.019
Non-institutional delivery	0.050	0.043	0.047	0.019	0.071	0.006	0.022	0.037	0.008	0.046
Using solid cooking fuel	-0.014	0.025	-0.096	0.015	-0.045	0.005	-0.019	0.021	-0.048	0.030
Belong to rural areas	0.044	0.021	0.054	0.005	0.062	0.000	0.027	0.018	0.042	0.021
Female child	0.032	0.100	0.031	0.009	0.045	0.004	0.030	0.086	0.038	0.084
Teen aged mothers	0.028	0.059	0.022	0.005	0.022	0.001	0.019	0.051	0.027	0.069
Having 3& more births	0.053	0.078	0.096	0.009	0.05	0.013	0.014	0.068	0.003	0.082
Average rainfall (°C)	0.000	0.005	0.030	0.008	0.030	0.003	0.000	0.004	-0.001	0.005
Average temperature (mm)	-0.291	0.070	-0.143	0.013	-0.112	0.009	-0.117	0.061	-0.106	0.089
R^2	0.17				0.54		0.29		0.31	
**AIC**	4953.57				1645.21		4810.00		4796.55	

Figure 1. (a) Spatial distribution of in-utero exposure to $PM_{2.5,}$ (b) low birth weight and (c) preterm birth across in India

Figure 2. Weighted percentage of low birth weight and preterm birth by background characteristics

Figure 3. Susceptibility to (a) low birth weight, and (b) preterm birth due to the in-utero exposure to PM2.5.

Figure 4. Bivariate LISA map showing the spatial association between in-utero exposure to $PM_{2.5}$ and low birth weight (a) and preterm birth (b).

Figure 5. Predicted low birth weight (LBW) and preterm birth (PTB) results from Ordinary List Square (OLS), Geographically Weighed Regression (GWR), Multiscale Geographically Weighed Regression (MGWR), Spatial Lag Model (SLM) and Spatial Error Model (SEM) after adjusting environmental, Socioeconomic maternal and child characteristics.

Click here to access/download;Figure;Figure 1.tif

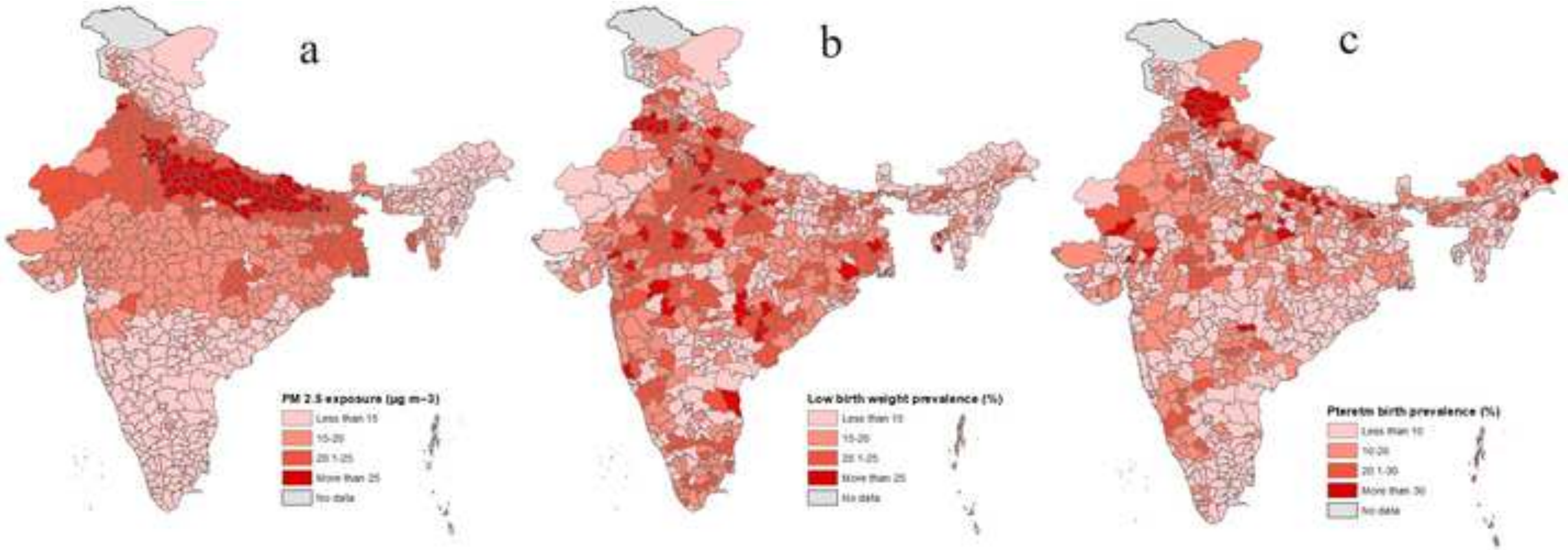

Click here to access/download;Figure;Figure 2.tif

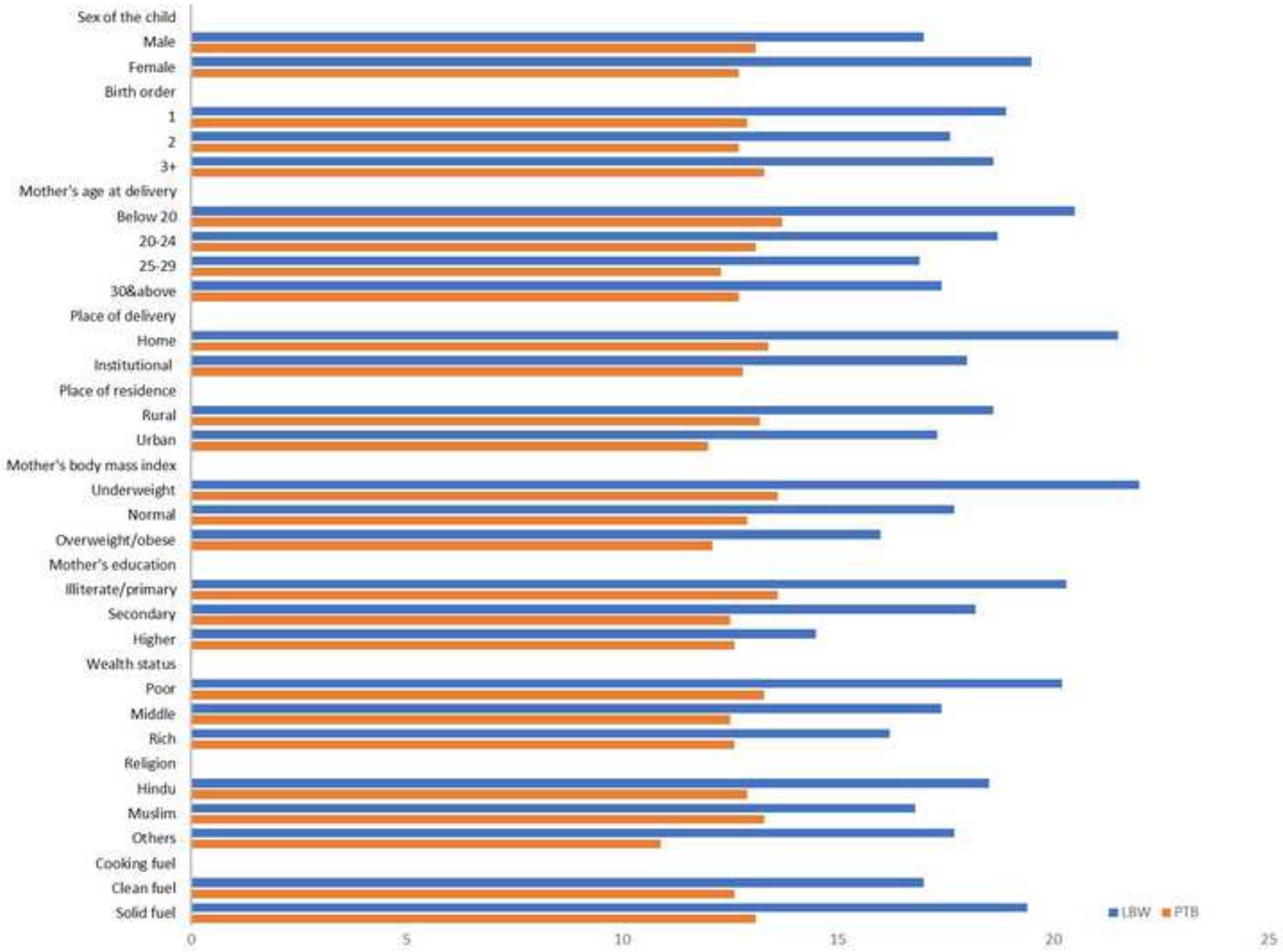

Click here to access/download;Figure;Figure 3.tif

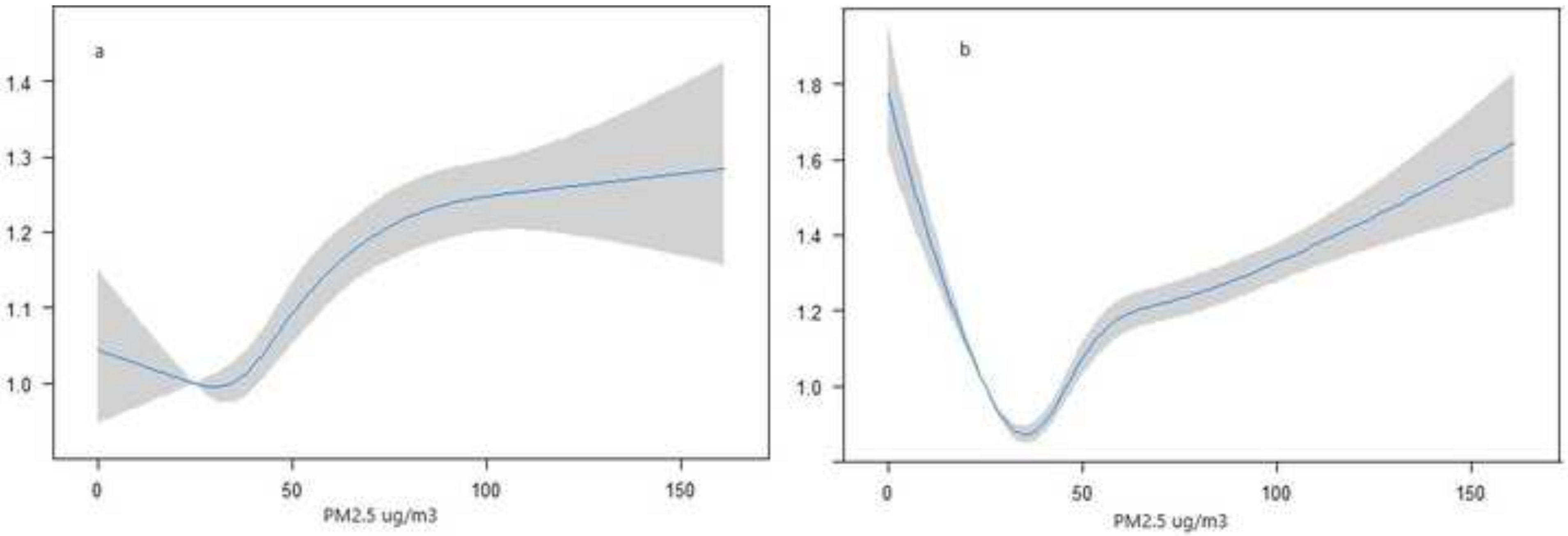

Click here to access/download;Figure;Figure 4.tif

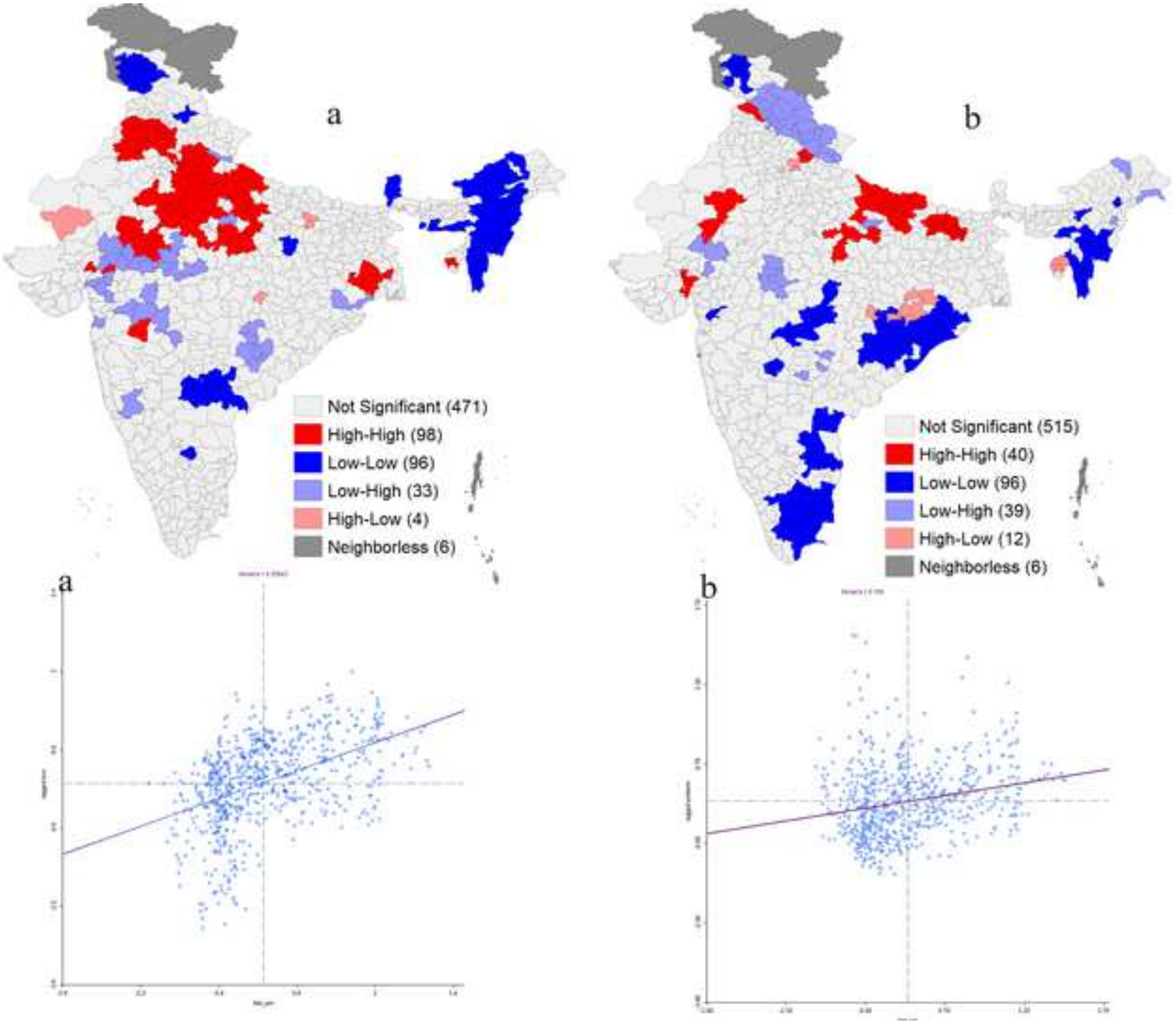

Click here to access/download;Figure;Figure 5.tif

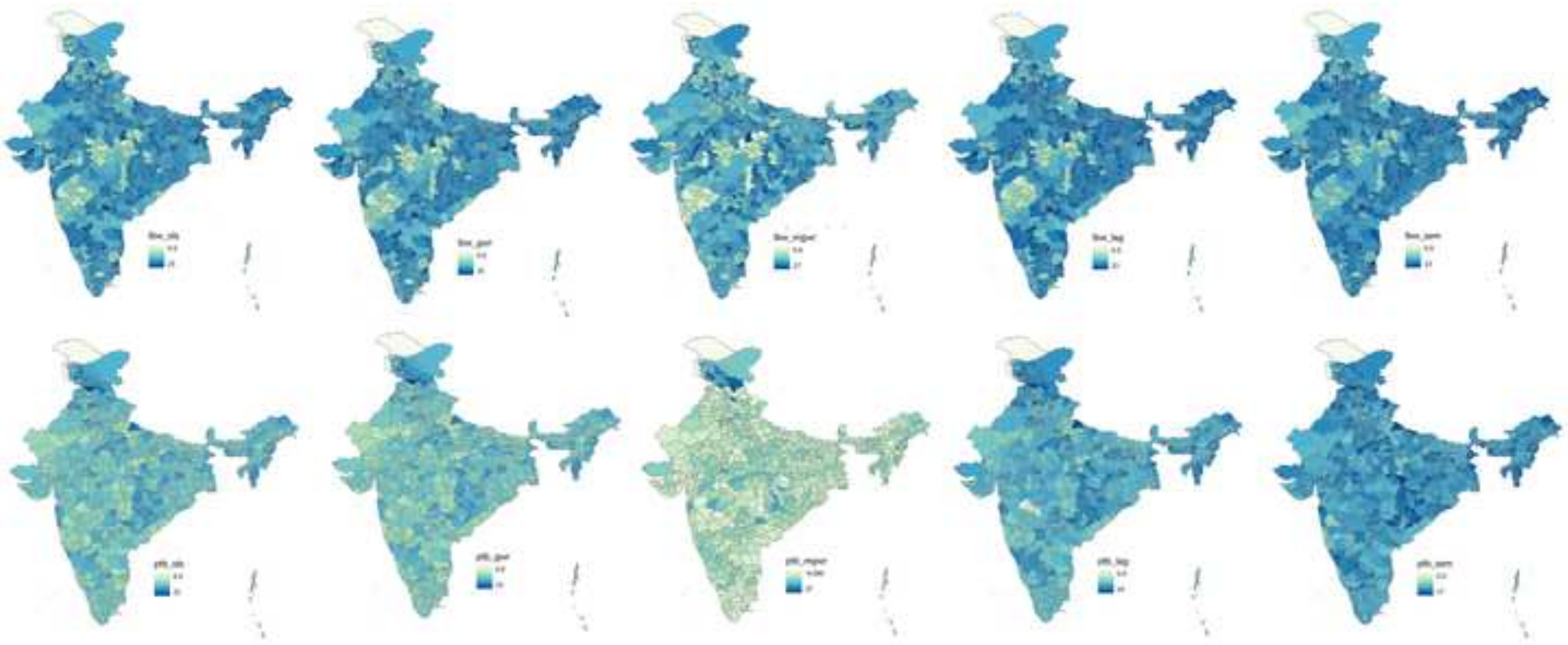

Click here to access/download
Supporting Information
Supplementary Material.docx
