# Supplementary material for: In-utero exposure to PM2.5 and adverse birth outcomes in India: Geostatistical modelling using remote sensing and demographic health survey data 2019–21
Source: PLOS Glob Public Health. 2025 Jul 2;5(7):e0003798. doi: 10.1371/journal.pgph.0003798 (PMC12220995; doi:10.1371/journal.pgph.0003798)
Supplement: Attachment — Submitted filename: response to reviewers.docx [file pgph.0003798.s004.docx]

Submission:
PGPH-D-24-02116
In-utero exposure to PM2.5 and adverse birth outcomes in India: Geostatistical modelling using remote sensing and demographic health survey data 2019-21
PLOS Global Public Health

Editor’s Comment

1. We noticed you have some minor occurrence of overlapping text with the following previous publication(s), which needs to be addressed:

https://www.nature.com/articles/s41370-023-00591-5’

https://www.researchgate.net/publication/341944202_Associations_between_green_space_and_preterm_birth_Windows_of_susceptibility_and_interaction_with_air_pollution

In your revision ensure you cite all your sources (including your own works), and quote or rephrase any duplicated text outside the methods section. Further consideration is dependent on these concerns being addressed.

Response: We have removed the plagiarism you mentioned and have properly cited the articles.

2. Figures 1, 4 and 5: please (a) provide a direct link to the base layer of the map (i.e., the country or region border shape) and ensure this is also included in the figure legend; and (b) provide a link to the terms of use / license information for the base layer image or shapefile. We cannot publish proprietary or copyrighted maps (e.g. Google Maps, Mapquest) and the terms of use for your map base layer must be compatible with our CC-BY 4.0 license.

Note: if you created the map in a software program like R or ArcGIS, please locate and indicate the source of the basemap shapefile onto which data has been plotted.

If your map was obtained from a copyrighted source please amend the figure so that the base map used is from an openly available source. Alternatively, please provide explicit written permission from the copyright holder granting you the right to publish the material under our CC-BY 4.0 license.

Please note that the following CC BY licenses are compatible with PLOS license: CC BY 4.0, CC BY 2.0 and CC BY 3.0, meanwhile such licenses as CC BY-ND 3.0 and others are not compatible due to additional restrictions.

If you are unsure whether you can use a map or not, please do reach out and we will be able to help you. The following websites are good examples of where you can source open access or public domain maps:

Response: We have generated all the maps mentioned in the revised manuscript.

Reviewer #1: Please refer to my detailed comments in the PDF version of the paper. My recommendation is to publish this article after revision. The findings and discussion section need significant improvement - improving the presentation of results in the findings section which is currently very "listy" and hard to follow for the reader, and the discussion section needs to significantly expand on the mechanisms and context driving the results.

Lots of acronyms - recommend using acronyms sparingly and not in the Abstract.

Response: Thank you for the suggestion. We have removed acronyms from the abstract.

The MGWR spatial model demonstrated the highest level of goodness of fit: more general language

Response: The sentence has been rewritten in simple language as suggested.

Alarmingly, in 2019, as many as **0.98 million deaths** in India were attributed to ambient particulate matter pollution. ??

Response: we have modified the sentence as “Study reported that about a million deaths in India were attributed to ambient particulate matter pollution in 2019”.

Here is seems like there is an established literature that air pollution -> negative health effects for infants and children. So, this paper's contribution is to confirm that - but more importantly it would be to provide evidence on the magnitude and factors that mitigate or exacerbate those outcomes.

Response: While LBW is a global public health concern, its prevalence is particularly higher in low and middle-income countries. A study reported that a 10 μg/m³ increase in PM2.5 reduced birth weight significantly in the first (−2.6 g) and third trimesters (−3.1 g). During the first trimester, PM2.5 exposure above 21.36 μg/m³ was significantly associated with higher odds of adverse outcomes (AOR: 1.29, 95% CI: 1.00–1.67). However, it has been reported by the previous studies that factors like access to prenatal care, improved nutrition, and air quality interventions, such as using air purifiers and limiting outdoor activity, can help mitigate these adverse effects.

Recommend tweaking the frame for the importance of this paper. This reads like the primary contribution is a test of the hypothesis that air pollution -> poor health outcomes. However as per all of the information above, this is relatively well established.

Response: Therefore, the study hypothesizes that there is a significant spatial variation in the impact of ambient air pollution on LBW and PTB across different regions of India, with specific areas showing higher vulnerability to these adverse birth outcomes due to elevated pollution levels.

According to the World Health Organization (WHO), **Preterm** birth is defined as a live birth before 37

176 completed weeks of gestation, while low birth weight is defined as weight at birth less than 2500 grams. Preterm

Response: Thank you. We have resolved the error.

The bivariate LISA functionality the cluster maps of the association between two variables.

This is not a complete sentence.

The cluster map is a special choropleth map showing those locations with a significant local Moran statistic classified by the type of spatial correlation: bright red for high-high associations, bright blue for low-low, light blue for low-high and light red for high-low.

This is not clear

The high-high and low-low suggest clustering of similar values, whereas high-low and low-high locations indicate spatial outliers. The scatter plot of the dependent and independent variables of a spatial unit shows the statistical figure of the association.

please clarify the methods discussion.

Response: Thank you for the suggestions. We have revised the manuscript as “The bivariate Local Indicators of Spatial Association (LISA) functionality is a powerful tool for exploring spatial relationships between two variables. It generates cluster maps that reveal the spatial association by identifying areas where the relationship between the two variables is statistically significant. A bivariate LISA cluster map is a specialized choropleth map that highlights spatial units with significant local Moran statistics, classifying them based on the type of spatial correlation observed. Specifically, bright red indicates high-high clusters, where high values of both variables are concentrated, and bright blue represents low-low clusters, where low values of both variables are grouped. Conversely, light red denotes high-low associations, where high values of one variable correspond to low values of the other, while light blue represents low-high associations, where low values of one variable are adjacent to high values of the other. High-high and low-low clusters suggest spatial clustering of similar values, while high-low and low-high clusters indicate spatial outliers, where neighboring values differ significantly. Additionally, the scatter plot of the dependent and independent variables for each spatial unit provides a statistical visualization of the association, complementing the cluster map by illustrating the nature and strength of the relationship between the two variables across the study area. Together, these tools enable a nuanced understanding of spatial patterns and their underlying associations, which is particularly valuable in geographic and spatial epidemiology studies.”

Several geostatistical models were used in this study to identify the best-fitting model, and the results will be helpful for policymakers to launch area-specific schemes for vulnerable regions. . . First

out of place in this section and perhaps an incomplete thought?

Response: Revision has been made as “Several geostatistical models were employed in this study to identify the best-fitting model for analyzing spatial patterns and associations. The results derived from this analysis provide critical insights that can guide policymakers in designing and implementing area-specific schemes, particularly targeting vulnerable regions identified through the models. These findings underscore the importance of evidence-based strategies for addressing regional disparities and improving resource allocation.”

In contrast, a lower prevalence of PTB was observed in Mizoram, Manipur and Tripura. As for LBW,

consistency please

Response: Revised as per the suggestion.

.3 Effects of PM2.5 on ABO at the individual level

This is an onslaught of descriptive statistics for the reader. Is there a way that this can be presented in a more compelling way/easier to follow and digest?

Response: we have revised the paragraph, made more concise and reader-friendly while preserving the descriptive statistics:

“Figure 2 illustrates the weighted percentage of LBW and PTB by selected background characteristics. The prevalence of LBW was higher among females (20%) compared to males (17%), while PTB showed no significant gender difference (Table S2). Teenage mothers exhibited the highest prevalence of both outcomes, with LBW rates decreasing with age but PTB rates rising after age 30. Children born at home or in rural areas had higher adverse birth outcomes. Underweight mothers had the highest rates of LBW (22%) and PTB (13%), compared to 17% and 12%, respectively, among normal-weight mothers. Educational and socioeconomic disparities were evident, as illiterate or less-educated mothers and poorer households had greater adverse birth outcomes. Mothers from Muslim households exhibited lower LBW rates but higher PTB prevalence. Additionally, households using solid fuels for cooking experienced higher rates of both LBW and PTB.”

Underweighted mothers had more LBW (22%) and PTB (13%) children than normal mothers, 17% and 12%, respectively. Illiterate and primary educated mothers were having more LBW and PTB children. A similar result can be observed for wealth status.

These are all correlated with poverty, correct?

Households using solid fuels for cooking had more adverse birth outcomes. I assume this is correlated with poverty.

A slight increase in temperature was associated with higher odds of LBW (AOR: 1.03; 95% CI: 1.01–1.04), though it was not significantly associated with PTB, while higher rainfall was significantly associated with both LBW (AOR: 1.07; 95% CI: 1.03–1.12) and PTB (AOR: 1.04; 95% CI: 1.02–0.10).

The climate relationship is more interesting to me because the poverty connection seems well-studied.

Response: Yes, there are studies exist they stated a connection of poverty, health outcomes and type of fuel use. In the present study, we do not have proper information to estimate poverty. We have used wealth index in the analysis which is a proxy of their economic status as the wealth index has been computed based on the assets reported in the survey by the respondents. And we found a relationship between ABOs and wealth status that discussed in the result section of the manuscript. However, we want to highlight one thing that in the present study we have used wealth status of the household along with other variables as a confounding factors though our prime objective is find out the connection between air pollution and ABOs.

There was a high likelihood of LBW in women who experienced high levels of PM2.5 during their gestational period

This seems to be a key finding but is buried after the other descriptive stats. I suggest reframing the presentation of results in this section.

Response: You're absolutely right, the finding of a strong association between high PM2.5 exposure during pregnancy and an increased risk of LBW is a key result of this study. We present the results in clarity for readers. This approach allows for a gradual understanding of the data, starting with the baseline characteristics of our study population, before moving on to the more complex statistical analyses.

This discrepancy underscores the influence of the various determinants considered in the study on the odds ratio values, highlighting their importance in the analysis. This is not clear to me.

Response: We have revised the para to make it simple as “The unadjusted analysis demonstrated a significantly increased risk of low birth weight (LBW) among women exposed to higher levels of PM2.5 during pregnancy (OR: 1.56, 95% CI: 1.50-1.63). However, after adjusting for a comprehensive set of confounders including environmental, socioeconomic, maternal, and child factors, the strength of this association attenuated. Conversely, for preterm birth (PTB), the adjusted odds ratio increased slightly compared to the unadjusted model. These contrasting findings emphasize the crucial role of rigorous adjustment for potential confounders in accurately assessing the impact of PM2.5 exposure on these adverse birth outcomes. These sensitivity analyses further strengthen the confidence in our findings, suggesting that the observed associations between PM2.5 exposure and adverse birth outcomes are robust.”

We observe a higher value of the odds ratio of having preterm infants whose mothers were exposed to PM2.5 during pregnancy as compared to the results for LBW. Why would this be the case?

Response: While our findings indicate a significant association between PM2.5 exposure during pregnancy and both low birth weight and preterm birth, the observed effect on preterm birth appears stronger. This discrepancy suggests that PM2.5 may exert distinct biological influences on these distinct pregnancy outcomes. Potential mechanisms include PM2.5-induced inflammation, disruption of fetal membranes, and placental dysfunction. However, further research is crucial to elucidate the specific pathways through which PM2.5 exposure differentially impacts these adverse birth outcomes.

Further, climatic factors such as rainfall and temperature are significantly associated with ABOs in India. Why? A correlation with the monsoon and solid fuel burning? More discussion of the mechanisms and context would improve the paper.

Response: Thank you for the comment. We have explained all the possible mechanisms of the effects of climatic factors on birth outcomes in the discussion section.

It is worth noting that except for Nagaland, North-Eastern states had a lower prevalence of preterm birth and low birth weight, coinciding with lower pollution levels in that region. Can you please describe why various geographies might have varying levels of these.

Response: We have added the explanations in the revised manuscript.

flipping between acronyms and the terms. I personally would prefer the terms given all of the other acronyms.

Response: Thank you for the comment. We have revised according to the comment.

The NFHS-5 report suggests that a large proportion of households in the Northern parts of India use solid fuels compared to other regions. It is well documented that the residential sector is a significant contributor to the total PM2.5 emissions along with the industry, energy and agriculture sectors. Among industrial, residential and energy sources, the contribution of energy sources to total emissions is the maximum, while residential sources contribute the maximum to PM2.5 emissions during winter and post-monsoon. Can you link/show this with your data?

Response: Establishing the link you mentioned is a complex task that extends beyond the scope of this paper. While this connection is well-documented by civil engineers and environmental scientists, it falls outside our area of expertise as social scientists. Although we have raised a valid query, we are unable to include this aspect in the current study

Moreover, the issue of air pollution is exacerbated by crop residue burning and forest fires in northern India, significantly contributing to the toxic air quality in the region. So is it the crop residue burning and forest fires or all of the other factors noted above that are driving the highest levels? Right now, this just reads as a list of potential mechanisms. Is it possible to map/investigate these with your data?

Response: The data used in this study does not include the requested information. It is important to note that a single study cannot address all possible mechanisms through its findings. Instead, we rely on findings from previous studies to explain these mechanisms in the discussion section, a common practice in scientific research. As our primary objective differs, and given the limitations of our data and expertise, we regret that we are unable to undertake this task. However, the statement we provided is grounded in well-established studies that we have already cited.

The mechanisms behind preterm birth due to exposure to PM2.5 are not clearly understood. Some epidemiological and toxicological studies proposed different pathways to explain the paradox. This paragraph should be at the beginning of the study in a background section.

Response: The paragraph has been added in the introduction.

An earlier study identified that maternal exposure to PM2.5 in the latter stages of pregnancy induces cytokine activation, favouring inflammation. Ibid comment above - this is background information that does not relate to your findings.

Response: We have removed the statement. All explanations provided in the discussion section focus solely on explaining the underlying mechanisms.

Sometimes inhaled PM2.5 penetrates the toxic gases that damage deoxyribonucleic acid (DNA), restricting nutrient supply… If a term will only be mentioned 1 or 2 times, my suggestion is NOT to use acronyms.

Response: Done.

Medical studies found that the mother's fetus grows rapidly in the third trimester of pregnancy. That period is more sensitive, and exposure to PM2.5 can hemorrhage the foetal growth easily. Nevertheless, the level of thyroid hormone might be affected by the toxicity of PM2.5, which is a responsible factor for less fetus weight. Previous studies have found that fetal growth depends on many factors, such as mothers' health, socioeconomic condition and genetic factors. Thus, the present study has adjusted for those potential plausible factors. Nevertheless, the study establishes a significant association between PM2.5 and LBW.

Ibid - this should be placed above.

Response: The revision has been made accordingly.

Low birth weight and preterm birth significantly contribute to the highest number of stunting and premature deaths in India. Either background or conclusion but this doesn't fit in the discussion of findings.

Response: The statement has been removed from the discussion.

Understanding the impact of exposure to indoor air pollution during pregnancy is also important because pregnant women tend to spend most of their time indoors and this time only increases once the pregnancy progresses. Do you have a citation for this?

Response: The citation has been added to support the statement.

In line with the hypothesis, our study also found a negative association between in-utero exposure to PM2.5 and birth weight, whereas the result for PTB was the opposite. This is not clear to me.

Response: Sorry for the confusing statement. We have revised the statement as “Understanding the impact of exposure to indoor air pollution during pregnancy is also important because pregnant women tend to spend most of their time indoors and this time only increases once the pregnancy progresses 106. Past studies found that releasing pollutants from uncleaned biomass burning in the household restricts fetal growth, increasing the probability of having a child with low weight and preterm birth 107,108. In line with the hypothesis, our study also found a negative association between in-utero exposure to PM2.5 and birth weight, whereas the result for PTB was not significant. However, addressing indoor air pollution through interventions such as promoting cleaner fuels and improving home ventilation is crucial for improving pregnancy outcomes and neonatal health.”

The present finds a significant role of climatic factors for ABOs. Previous evidence suggests that high temperatures can cause heat stress, dehydration, and reduced uteroplacental… The study

Response: Revision has been made.

Our findings suggest that both indoor and ambient air pollution along with climatic factor are significantly associated with ABOs. But you just have one question in the survey on burning solid fuels and you don't have any actual measures of indoor air pollution levels, right? There are a number of studies that have these indoor/localized measures. I would just recommend keeping your paper focused on ambient air pollution given your data/methods versus putting a lot of emphasis on the indoor air pollution finding.

Response: Thank you for the comment. We have removed the detailed discussion on indoor air pollution in the revised manuscript.

that mothers did not change their residence during the period from pregnancy to the conducting date of the survey. The study acknowledges that like some other possible determinants, smoking practices and alcohol consumption were not considered in the model due to the under-reporting nature. Also, the possibility of biases due to self-reporting cannot be ignored. Due to the cross-sectional nature of the study, we cannot draw a causal relationship, other aspects including the seasonality effects can be explored using the longitudinal study in the future. As India is not satisfactorily progressing in child nutrition, hence consorted effort to improve environmental conditions, especially air quality, is essential to tackle issues related to child health.

This should be a separate paragraph and my suggestion is to put this earlier in the paper - perhaps a limitations section in the Methods section?

Response: This paragraph highlights the limitations of the study. To address potential confusion among readers, we have added a heading titled “Limitations” before the conclusion section in the revised manuscript.

5. Conclusion

Anything about how the methods could be replicated/expanded/combined with other datasets?

Response: Yes, the method can be replicated with other datasets but depends on the research questions and the nature and quality of the data. We have added one statement in the conclusion – “The geostatistical models can be utilized in future studies to support area-specific policy development by identifying zones that are more vulnerable.”

Reviewer #2: General comments:

The manuscript presents a significant and timely topic on examining the relationship between PM2.5 air pollution and adverse birth outcomes (ABOs) in India. The study employs robust data from the National Family Health Survey (NFHS) combined with satellite-based PM2.5 and climatic data, offering a unique geostatistical approach to identify vulnerable populations at individual and district levels. The findings—highlighting associations between PM2.5 exposure and higher risks of low birth weight (LBW) and preterm birth (PTB)—are compelling and policy-relevant. However, the manuscript has some methodological and interpretive limitations that must be acknowledged in the Discussion before it can be considered for publication.

Response: Thank you so much as appreciating the relevance of the paper and giving us the comments and suggestions that will definitely helpful to improve the quality of the paper.

Major comments:

1. The methods section does not specify whether non-linear relationships between PM2.5 exposure and birth outcomes were considered. If only linear models were used, the analysis may fail to capture critical thresholds at very high or low exposure levels. This should be included in the discussion as a potential limitation.

Response: We have used both linear and nonlinear relationship as well. For example, we have used the polynomial function in the study which is mentioned in the method section “Further, in the study, a cubic spline function was utilized to visually depict the connection. This connection involved the likelihood of occurrences such as having children with LBW and PTB, all influenced by exposure to air pollution during pregnancy. A spline function integrates several polynomial sections linked by knots, forming a continuous curve. The quantity of knots can be modified to control the curve and smoothness, increasing or decreasing it as needed. To determine knot placement, we employed quantiles of the exposure level of PM2.5 in the individuals.”

2. The reliance on self-reported birth outcomes in NFHS may introduce recall bias. This should be acknowledged as another potential limitation.

Response: Thank you, we have added the limitation in the revised manuscript as “Additionally, the potential for biases arising from self-reported birth weight and pregnancy duration cannot be ignored”.

3. The study uses annual average PM2.5 concentrations derived from satellite data. This approach does not account for within-year variations in exposure, such as seasonal fluctuations, which could influence pregnancy outcomes. The authors should discuss how this temporal resolution might misclassify exposure, particularly in areas with distinct pollution patterns across seasons.

Response: Monthly PM2.5 data was utilized in the study, capturing monthly variations during exposure calculation. However, seasonal variations could not be analyzed, as the average pregnancy duration is nine months, and only in-utero exposure was considered to assess PM2.5’s effects on birth outcomes.

4. While temperature and rainfall are included, the methods do not clarify whether these variables were spatially or temporally aligned with PM2.5 exposure or birth outcome data. Mismatched temporal or spatial aggregation could introduce exposure misclassification and bias the findings. Additional details should be provided and discussed in detail.

Response: In the study, all exposure measures, including PM2.5, temperature, and rainfall, were calculated for the entire pregnancy period of each individual. Methodologically, the spatio-temporal aspects of all exposure variables are accurate. We have included additional details in the Methods section in response to the comment. As our prime objective is to explore the effects of PM2.5, we have not added the temperature and rainfall exposure maps.

5. Although the authors mention smoking and alcohol use as unmeasured confounders, they do not acknowledge other potential behavioral confounders, such as maternal physical activity, diet, and access to prenatal care, which may vary spatially and interact with air pollution exposure. Please include these.

Response: Yes, you are right that these variables are important determinants of ABOs. However, the DHS survey does not collect this information. Although antenatal visit data is available, it is only for the last birth. Therefore, we acknowledge this as a limitation of our study, and it has been included in the limitations section of the revised manuscript.

Minor comments:

6. Across the manuscript, please mention that the observed effects are per unit increase in PM2.5.

Response: The values are there in the last para of the results that point we have added in the abstract too. Thank you for pointing out.

7. In the abstract, please mention the abbreviations in full as they first appear in text: LBW, AOR, PM2.5, MGWR.

Response: the amendment has been done.

8. Effects of rainfall and temperature are only briefly mentioned in abstract. These should be expanded to provide the full context of these findings.

Response: Thank to for the comment. We have added a detailed explanation in the revised manuscript.

9. Correct the “mu” symbol in the unit of measurement of PM2.5 throughout the manuscript.

Response: the amendment has been done.

10. Lines 98-99 in the Introduction suggest that only LBW and PTB have been associated with air pollution in the existing literature. There are other adverse birth outcomes such as spontaneous abortion for which epidemiological evidence exists. Please correct the sentence to be more inclusive.

Response: The amendment has been done.
